# Plastic and terrestrial organic matter degradation by the humic lake microbiome continues throughout the seasons

**DOI:** 10.1111/1758-2229.13302

**Published:** 2024-06-09

**Authors:** Jussi S. Vesamäki, Miikka B. Laine, Riitta Nissinen, Sami J. Taipale

**Affiliations:** ^1^ Department of Biological and Environmental Science University of Jyväskylä Jyväskylä Finland; ^2^ Department of Biology University of Turku Turku Finland

## Abstract

Boreal freshwaters go through four seasons, however, studies about the decomposition of terrestrial and plastic compounds often focus only on summer. We compared microbial decomposition of ^13^C‐polyethylene, ^13^C‐polystyrene, and ^13^C‐plant litter (*Typha latifolia*) by determining the biochemical fate of the substrate carbon and identified the microbial decomposer taxa in humic lake waters in four seasons. For the first time, the annual decomposition rate including separated seasonal variation was calculated for microplastics and plant litter in the freshwater system. Polyethylene decomposition was not detected, whereas polystyrene and plant litter were degraded in all seasons. In winter, decomposition rates of polystyrene and plant litter were fivefold and fourfold slower than in summer, respectively. Carbon from each substrate was mainly respired in all seasons. Plant litter was utilized efficiently by various microbial groups, whereas polystyrene decomposition was limited to Alpha‐ and Gammaproteobacteria. The decomposition was not restricted only to the growth season, highlighting that the decomposition of both labile organic matter and extremely recalcitrant microplastics continues throughout the seasons.

## Graphical abstract

We explored the seasonal variation in the microbial degradation of microplastics and plant litter in humic lake waters. Seasonal changes affected decomposition rates, carbon cycling, and microbial communities behind the degradation. Both plant litter and recalcitrant plastic were degraded in all seasons, revealing that the decomposition continues throughout the year.

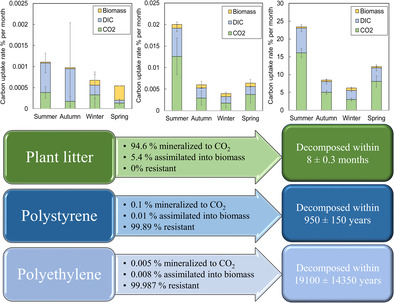

## INTRODUCTION

Microbes play a crucial role in decomposition and carbon cycling processes in lentic freshwaters, where microbes dominate plant litter decomposition over aquatic shredders, bringing carbon, nutrients, and energy from terrestrial organic matter (OM) available for aquatic consumers (Attermeyer et al., 2013; DeGasparro et al., 2020; Raposeiro et al., 2017). As an important source of carbon, nutrients, and energy, the input of terrestrial OM supports the aquatic food web and is a precursor for humus formation (Danise et al., 2018; Kritzberg et al., 2004; Taipale et al., 2023). It enters lake water via soil runoff and OM (e.g., plant litter) input, shaping microbial community succession, and increasing microbial biomass production and activity (Attermeyer et al., 2013; Hutalle‐Schmelzer et al., 2010). In addition to natural sources of terrestrial OM, varying concentrations (0.27–34,000 particle/m^3^) of microplastic particles (diameter <5 mm) have been found in freshwater lakes worldwide, mostly as fibres (Dusaucy et al., 2021; Uurasjärvi et al., 2020). Due to increasing plastic pollution in all ecosystems, including oceans (Jambeck et al., 2015) and freshwaters (Hurley et al., 2018), microplastic pollution has become a global environmental concern. They are highly persistent and can remain in the water column for a long time, accumulate in organisms or sediments, and reduce the decomposition rate of OM (Bertoli et al., 2023; Welsh et al., 2022). Despite their potentially harmful effects on ecosystems, recent studies have shown that carbon from plastic can be nutritionally upgraded in the lake food web via microbial decomposition, suggesting that even highly persistent microplastic carbon can subsidize aquatic food web and be brought to fast carbon cycling (Taipale et al., 2019, 2023). Therefore, plastics represent a novel source of terrestrial OM for aquatic ecosystems (Hoellein et al., 2019; Vincent & Hoellein, 2021). Particularly in humic lakes (defined often as dissolved organic carbon [DOC] >10 mg/L) degradation of microplastics is faster than in clear lake waters (Taipale et al., 2019, 2022; Vesamäki et al., 2022). Humic lakes play an important role in global carbon cycling due to their role as sources of carbon emissions (Kiuru et al., 2018; Sobek et al., 2006), high occurrence in a boreal zone (Kortelainen, 1999; Rantakari et al., 2004), and increasing number as a result of brownification (Blanchet et al., 2022; Williamson et al., 2020). Despite the growing impact of humic lakes on global carbon cycling, the effect of seasonal variation in degradation processes of plastics and OM in humic freshwaters have been overlooked.

In boreal lakes which go through four different seasons, seasonality is shaped by simultaneous changes in several environmental parameters, including, for example, temperature, nutrient content, pH, oxygen concentration, and OM input from catchment (Fuentes et al., 2013; Hanson et al., 2006; Kreeger et al., 1997; Mihu‐Pintilie et al., 2014). These environmental parameters further drive changes in organism growth (Bernal‐Brooks et al., 2003; Neuenschwander et al., 2015; Vinni et al., 2004), lake autotrophy (Laas et al., 2012; Sanders et al., 1990), microbial community composition (Duarte et al., 2016; Lew et al., 2015; Newman et al., 2015), and decomposition processes (Liu et al., 2022; Mishra et al., 2022; Shaji et al., 2024; van Dokkum et al., 2002). For instance, a temperature that is higher in summer is known to accelerate microbial decomposition and activity, which may be further fostered by simultaneously elevating nutrient availability (Duarte et al., 2016; Fernandes et al., 2012). Since seasonal changes affect both decomposition and microbial community composition (Duarte et al., 2016; Newman et al., 2015), it raises the question if substrate decomposers vary seasonally as well. In lake water, which has a variety of different carbon sources, decomposers of plastics and OM cannot be determined examining only microbial taxa that are growing on substrate or become more general, since these may utilize other available carbon sources and use added particles only as a physical growth substrate. Combining ^13^C‐isotope analysis with phospholipid fatty acid (PLFA) biomarker and microbial community analyses, involvement of microbial taxa in the decomposition process can be more accurately traced (Taipale et al., 2019, 2022; Twining et al., 2020). In addition, this allows to follow the biochemical fate of added substrate carbon after microbial processing (Steffens et al., 2015; Taipale et al., 2023; Vesamäki et al., 2022; Yang et al., 2015). The fate of carbon is defined as the endpoint of the carbon; carbon can be mineralized to CO_2_ via respiration or used as an organic compound for anabolic processes, thus assimilated into microbial biomass (Liu et al., 2021; Yuan et al., 2020). Despite the importance of terrestrial subsidy to the aquatic carbon budget (Sobek et al., 2006), food webs (Brett et al., 2017; Taipale et al., 2023), and the lake heterotrophy (Berggren et al., 2012; Laas et al., 2012), the fate of terrestrial carbon after microbial decomposition in freshwaters remains poorly understood (Freeman et al., 2024).

The present study aimed to determine how seasonal changes affect the microbial decomposition of labile versus recalcitrant carbon sources and the fate of substrate carbon in freshwaters. Therefore, we compared seasonal differences in microbial decomposition processes of three substrates with contrasting chemical structures and recalcitrance in three highly humic lake waters collected in spring, summer, autumn, and winter. Studied substrates included two types of ^13^C‐labelled microplastics (aliphatic polyethylene [PE] and aromatic polystyrene [PS]) and ^13^C‐labelled plant litter (*Typha latifolia*). Plant litter was selected to represent a labile organic carbon source that is efficiently utilized by microbes, whereas plastics represented a recalcitrant carbon source. Both PE and PS represent a common plastic type found in lakes (Dusaucy et al., 2021; Uurasjärvi et al., 2020). Studied plastics differ by their chemical structure which affects their decomposition rate: aromatic PS is more prone to biodegradation than aliphatic PE (Debroas et al., 2017; Vesamäki et al., 2022). Despite differing decomposition rates, both aromatic and aliphatic plastics are rather used for biomass than respiration in summer (Vesamäki et al., 2022). To study the effects of seasonal variation on the decomposition process (the decomposition rate, the fate of carbon, and decomposer taxa), the following hypotheses were tested: (1) decomposition rates of microplastics and plant litter vary seasonally and are higher in summer than in other seasons, (2) microbial decomposer taxa vary seasonally, and (3) carbon from leaves is rather assimilated into biomass than respired in all seasons, whereas plastic carbon is mainly respired.

## EXPERIMENTAL PROCEDURES

### Sampling sites and experimental setup

Waters were collected from pelagic zone of three humic lakes called Lake Haukijärvi (61°22′29″ N, 25°13′79″ E), Lake Majajärvi (61°21′49″ N, 25°13′68″ E), and Lake Nimetön (61°22′82″ N, 25°19′26″ E) in Evo national park (Finland, Hämeenlinna) in 31 July 2021 (summer lake waters), 29th of October 2021 (autumn lake waters), 19 January 2022 (winter lake waters), and 17 May 2022 (spring lake waters). All lakes were surrounded by a mixed forest. Collected waters were filtrated through a 3 μm pore size filter to remove bacterivores after which 150 ml of lake water was poured into a 240 ml glass bottle and 2 mg C of ^13^C‐substrate (PE (Poly(ethylene‐^13^C_2_) Sigma‐Aldrich, 99 atom% ^13^C, USA), PS (Poly(styrene‐α‐^13^C) Sigma‐Aldrich, 99 atom% ^13^C, USA), or ^13^C‐plant litter (*T. latifolia*)) was added. The chosen substrate concentration was selected based on previous studies and the concentration of microplastics detected from lake water columns (Dusaucy et al., 2021; Taipale et al., 2019, 2022; Vesamäki et al., 2022).

Four experiments (one for each season) were conducted in three different humic lake water all of which had four treatments (PE, PS, and plant litter addition, and control without any substrate addition). Four replicates were made for each treatment. Bottles were incubated in darkness for 4 weeks at either 21°C (summer), 8°C (autumn), 2°C (winter), or 15°C (spring) and shaken daily. The total number of bottles was thus 48 per season (three lakes, four treatments, four replicates), and the total number of samples from all experiments was 192.

### Water parameters

In the field, temperature and oxygen concentration were measured from the whole water column with YSI3000. Lake water pH was measured with PHM220 Lab pH Meter, MeterLab™. The device was calibrated using a KCl solution at pH 4 and pH 7.

Concentrations of DOC and total nitrogen (TN) were measured by the Shimadzu TOC‐V cph total organic carbon analyser. For analysis, 20 ml subsample of water was filtered (Sartorius 0.45 μm pore size) and 80 μl of 2 M HCl was added. A standard curve with known concentrations of carbon and nitrogen diluted with deionized H_2_O was created for the quantification of DOC and TN.

The ascorbic acid method was used for the quantification of total phosphorus (TP) concentrations. Then, 500 μl of 4 M H_2_SO_4_ was added to a 50 ml filtrated lake water sample (Sartorius 0.45 μm pore size). TP was measured spectrometrically at 880 nm (Ordior UV‐1800 Spectrophotometer, Shimadzu).

### Microbial respiration

Gas samples were collected from the air phase of the bottle three times per week to follow CO_2_ and CH_4_ production in bottles. Then, 5 ml of gas sample was transferred into an air‐free Exetainer® tube after which the amount of CO_2_ and CH_4_ was determined by an Agilent 7890B gas chromatograph (Agilent Technologies, Palo Alto, CA, USA). At the end of the experiment, dissolved inorganic carbon (DIC) was analysed by taking 5 ml of water into a He‐flushed Exetainer® tube with 200 μl of 85% H_3_PO_4_ (Taipale & Sonninen, 2009). Water samples were mixed by a vortex and 5 ml of the gas phase was taken from the Exetainer® tube into a new tube. The gaseous DIC samples were further processed and analysed identically to air phase samples.

Concentrations of carbon in the gas and water phases were calculated. Since the measurement temperature and experimental temperatures were different, Charles's law was used to correct the change of the sample volume to get the standardized volume (5 ml at the RT) for each sample. After the correction, the amount of substance (n) in gas (sample + He) as mols were calculated: n(gas) = (V_tube_ + V_sample_)/V_m_, where V_tube_ is the volume of the Exetainer® tube (L) and V_sample_ is the gas sample volume (L), and V_m_ is the molar volume of ideal gas as L/mol. Then, we calculated the amount of CO_2_ (mol) from this gas mixture: n(CO_2_) = TCD/100,0000 × n(gas), which was further multiplied with a CF to get an actual amount of CO_2_ in the sample tube (thus, per 5 ml of gas): CF = (V_tube_ + V_sample_)/V_sample_. Then, the amount of substance was converted to mass (g): m(CO_2_) = n(CO_2_) × M_CO2_, where M_CO2_ is the molar mass of CO_2_ (g/mol). The proportion of the carbon from the total mass of CO_2_ in the sample was then determined as follows: m(C) = m(CO_2_) × (M_C_/M_CO2_), where M_C_ is the molar mass of carbon. At this point, the result was grams of C per 5 ml of the gas phase, and thus we calculated the concentration per litre as: c(C) = m(C) × (1000/5), which was further multiplied with 1000 to get a value as mg/L.

δ^13^C values of CO_2_ and DIC were analysed using an Isoprime TraceGas pre‐concentrator unit connected to an Isoprime IRMS (Isoprime100 IRMS, Elementar UK Ltd., Cheadle, UK) as described in the previous study (Vesamäki et al., 2022). δ^13^C values were drift corrected and two‐point calibrated based on external standards.

### Phospholipid‐derived fatty acid analysis and bulk PLFA‐SIA

Total of 100 ml of lake water from each bottle was filtrated after the 4‐week incubation period for microbial PLFA analysis through preweighed filters (Whatman™ cellulose nitrate filters, pore size 0.2 μm, diameter 47 mm). Filters were stored at −80°C, freeze‐dried, and weighed. Weighed filters were placed into a Kimax® tube with 3 ml of chloroform‐methanol (2:1). Then, 750 μl distilled water and internal standards PLFA C12:0, PLFA C19:0, and C23:0 (0.4995 mg/L, 0.5015 mg/ml, and 0.5007 mg/ml, respectively) were added into a sample tube. Lipid materials were extracted by applying Folch's method (Folch et al., 1957). Sample tubes were sonicated for 10 min after which they were centrifuged (3000 rpm; 4 min). The separated lower phase was transferred to a new tube. To maximize the lipid recovery, the phase separation was made twice. The second extraction was done by adding 2 ml of chloroform to the samples after the first extraction and repeating the sonication and centrifugation similarly to the first extraction. The extracted lipid phases were then evaporated to complete dryness under the N_2_ stream. Subsequently, lipids were fractioned via solid phase extraction using silica cartridges (500 mg, Agilent). The cartridges were activated with 3 ml of 1:1 chloroform:methanol solution, and samples were washed into cartridges with 400 μl of chloroform. Neutral lipids and glycolipids were eluted and discarded with 8 ml of chloroform and acetone. PLFAs, in turn, were eluted and collected into new tubes with 8 ml of methanol.

For analysis of the δ^13^C value of the bulk PLFA, PLFA‐fractions were evaporated to complete dryness under the N_2_ stream and 300 μl of CHCl_3_ was added. Then, 100 μl subsample was transferred into a pre‐weighed tin cup and evaporated. The rest of the sample was stored at −20°C until transesterification. Chloroform was evaporated and the tin cup was weighed. δ^13^C of the bulk PLFA sample was measured with a Thermo Finnigan DELTA^plus^Advantage CF‐IRMS, and drift corrected using external standards.

The transesterification of fatty acid methyl esters (FAMEs) was made by evaporating the remaining 2/3 subsample of PLFA‐fractions to complete dryness under N_2_ stream and adding 1 ml of toluene and 2 ml of 1% (v/v) sulphuric acid methanol solution. The transmethylation was made by keeping the samples on heat blocks at 90°C for 90 min. The FAMEs were extracted by adding 2 ml of MQ‐water and n‐hexane to the samples and centrifuging (2000 rpm, 2 min) them. The upper phases were then transferred to new tubes, evaporated to dryness under the N_2_ stream, washed twice into clear glass GC V‐vials with n‐hexane, and the excess n‐hexane was evaporated. The final volume for the samples was set at 80 or 100 μl with n‐hexane, and the samples were stored at −20°C until analysis.

FAMEs were analysed with a gas chromatograph (Shimadzu Ultra, Kyoto, Japan) equipped with a mass detector (GC–MS) using helium as the carrier gas (linear velocity = 36.3 cm/s). The injection temperature was 260°C, and the injection mode used was splitless (for 1 min). The interface and ion source temperatures used were 250 and 220°C, respectively. For batches A–C, an Agilent (Palo Alto, CA, USA) DB‐23 column (length 65 m, diameter 0.25 mm, film thickness 0.25 μm) was used with the following temperature program: 60°C was held for 1 min, then the temperature was increased to 130°C at 30°C/min rates, followed by 7°C/min rate increase to 180°C, and 1.5°C/min rate increase to 220°C where it was held for 10 min. In turn, for batch D, an Agilent DB‐Fast FAME column (length 30 m, diameter 0.25 mm, film thickness 0.25 μm) was used with the following temperature program: 60°C was held for 1 min, then the temperature was increased to 165°C at 40°C/min rate, followed by 4°C/min rate increase to 230°C, where it was held for 4.5 min. The total program times were 47.11 and 25.38 min, respectively, and the solvent cut time was 6 min.

The FAMEs were identified by using specific target ions and their retention times (RT) (Taipale et al., 2016). We applied four‐point calibration curves (15, 50, 100, and 250 ng/μl) based on a known standard solution of a FAME standard mixture (GLC standard mixture 566c, Nu‐Chek Prep, Elysian, MN, USA) to calculate the concentrations for the individual FAMEs in the samples. The quantifications were made with GCMS solution software (v4.42, Shimadzu, Japan) and the Pearson correlation value of the calibration curves for each FAME in the standard mixture was >0.99. Recovery of PLFAs was corrected based on the internal standard and the amount of PLFAs in a sample was calculated as mg/g of carbon. Since 4% of bacterial biomass is composed of PLFAs, the total PLFA content of the sample was multiplied by 25 to calculate the total microbial biomass (Taipale et al., 2015).

The δ^13^C values of each PLFA were measured with a 5977B GC/MSD (Agilent) coupled with the Isoprime PrecisION (Elementar) isotope ratio mass spectrometer at the University of Jyväskylä. PLFAs were separated using a 60 m DB‐23 column (diameter 0.25 mm, film thickness 0.25 μm) and oxidized to CO_2_ in an oxidation reactor at a temperature of 940°C with the reduction reactor kept at 630°C as described previously (Pilecky et al., 2023; Taipale et al., 2023). External standard F8.3 was used as a standard for drift correction and peak integration. Only peaks whose height (nA) was >0.015 were included in further analysis.

### Mineralization, assimilation, and decomposition rates and bacterial growth efficiency

δ^13^C values of CO_2_, DIC, and biomass were converted to atomic percentage (AP) using the equation (Fry, 2006): AP = (δ^13^C + 1000)/(δ^13^C + 1000 + 1000/R_standard_) × 100, where R_standard_ is value 0.01118 (VPDB). The control AP average was subtracted from the sample AP values (ΔAP) and divided by 100 to quantify the difference between control and sample AP values as decimals. Mineralization into CO_2_ or DIC and the assimilation rate were then separately calculated as % per day: (ΔAP × m_CO2/DIC/biomass_/m_added13C_ × 100)/t_exp_, where m_CO2/DIC/biomass_ is the mass of CO_2_, DIC or biomass, m_added13C_ is the mass of added ^13^C‐carbon, and t_exp_ is the incubation time as days. Further, the total mineralization rate of ^13^C‐substrates was calculated as the sum of the uptake rate into DIC and CO_2_. The decomposition rate was calculated as a sum of mineralization and assimilation rates. Decomposition rates per month were calculated for each substrate. Then, annual decomposition was calculated from monthly decomposition rates as follows: decomp_summer_ × 3 + decomp_autumn_ × 3 + decomp_winter_ × 3 + decomp_spring_ × 3, thus assuming an equal duration of each season. Bacterial growth efficiency (BGE) was calculated by dividing the assimilation rate by the total decomposition rate and multiplying the results by 100 (del Giorgio & Cole, 1998).

### Active bacterial communities

For microbial community samples, 20 ml of lake water from each bottle was filtrated at the end of each experiment (Supor® 0.2 μm/25 mm, PES, Pall Corporation). Filters were immediately transferred into a bashing bead lysis tube with 800 μl of DNA/RNA Shield™ and stored at −80°C until RNA extraction. RNA was extracted using a Chemagic™ 360 and the Chemagic™ Viral DNA/RNA 300 Kit H96 following the manufacturer's instructions (PerkinElmer, Waltham, MA, USA). For each sample, one aliquot of RNA was treated with DNAse and reverse transcribed to cDNA using the Maxima First Strand cDNA Synthesis Kit (Thermo Fisher Scientific, Waltham, MA, USA), after which cDNA samples were stored at −20°C. The effectiveness of the DNase step was validated by including negative RT samples.

The target region of the bacterial 16S SSU rRNA was amplified using the primer pair 515F–806R (Caporaso et al., 2011; Parada et al., 2016), to which were added the M13 linker (to the forward primer 515F) and the P1 adapter (to the reverse primer 806R). The first polymerase chain reaction (PCR) was done in a total volume of 25 μl: 12.5 μl of Maxima SYBR Green/Fluorescein qPCR Master Mix (Thermo Fisher Scientific), 9 μl of sterile water, 0.75 μl of both the forward and reverse primers (working solutions at 10 μM) and finally 2 μl of template cDNA. The amplification protocol included the following steps: initial desaturation of 3 min at 95°C, followed by 35 cycles of 45 s at 95°C, 1 min at 50°C and 90 s at 72°C, and a final extension of 10 min at 72°C. No amplification was observed in the negative RT samples or the NTC (no template control).

The second PCR, for barcoding, was done using a volume of 25 μl for each sample but using only 1 μl of the template (product of the first PCR), 0.75 μl of the reverse primer 806R‐P1 (working solution at 1 μM), 12.5 μl of DreamTaq Green PCR Master Mix (Thermo Fisher Scientific) and 10 μl of sterile water. The forward primers were the M13‐tailed Ion Torrent™ barcodes which were added individually to each reaction (working solution 10 μM). The amplification protocol was the same as during the first PCR but only consisted of 10 cycles.

After the barcoding step, each sample was purified using the SparQ PureMag Beads (Quantabio). Samples concentrations were measured using a Qubit fluorometer (Invitrogen/Thermo Fisher Scientific). Then, 10 ng of each sample was pooled together, and the pool was purified again. The pool quality and molarity were checked using a TapeStation 2200 and the High Sensitivity D1000 ScreenTape and reagents (Agilent). The sequencing was performed with the Ion Torrent Personal Genome Machine (Thermo Fisher Scientific) using the Ion PGM Hi‐Q View OT2 400 kit, the Ion PGM Hi‐Q View Sequencing kit (quality control included), and the Ion 318v2 chip. The sequences were then analysed using the CLC Genomics Workbench software (Qiagen). The primers were trimmed, and the short sequences were discarded (<150 bp). After the sequences were trimmed to the same length (average length: 252.8 bp; the number of reads after trimming: 22000 ± 13,000 reads; rarefied at the depth of 4000 based on the minimum number of observed reads in one sample), the OTU clustering was performed using the SILVA 16S v132 database with a similarity percentage of 99%. Raw sequence reads were deposited to the NCBI Sequence Read Archive.

### Statistical analysis

Statistical analyses were conducted using the software Primer 7. Differences in single environmental parameters, concentration of TIC, biomass, mineralization rate, assimilation rate, decomposition rate, BGE, and the comparison of δ^13^C values of each PLFA were tested separately with univariate analysis of variance (ANOVA) applying permutational multivariate ANOVA (PERMANOVA) on Euclidean distance matrices for single variables as described previously (Taipale et al., 2023). Monte Carlo's simulations were used for *p*‐values as suggested for data with a low number of replicates (Anderson & Robinson, 2003). Results were considered statistically significant if *p* < 0.05.

OTUs with relative abundance >0.5% of all detected 16S rRNA sequence reads were included for further analysis and the contribution of each OTU as % was used for statistical analysis. Differences in microbial community structures were tested with PERMANOVA after the square‐root transformation of data and calculation of the Bray–Curtis similarity matrix. Analysis of similarity percentages (SIMPER) was further conducted to identify microbial classes responsible for differences in community structures between studied substrates and seasons. The comparison of microbiome community structures between the controls and plant litter treatments was conducted by SIMPER to recognize microbial genera that participated in carbon recycling. Nonmetric multidimensional analysis (nMDS) with hierarchical cluster analysis were combined to analyse and visualize the clustering of treatments based on microbial community data and environmental variables (Clarke, 1993). Microbial genera associated with the most efficient substrate decomposition within each season were recognized by Spearman correlations (>0.6) and visualized with nMDS. Additionally, Pearson correlations and *R* ^2^‐values between the relative abundance of each microbial genus and the decomposition rate of PS were determined to exclude taxa whose participation in the decomposition was more unlikely.

## RESULTS

### Seasonal variation of lake water chemistry

**FIGURE 1:**
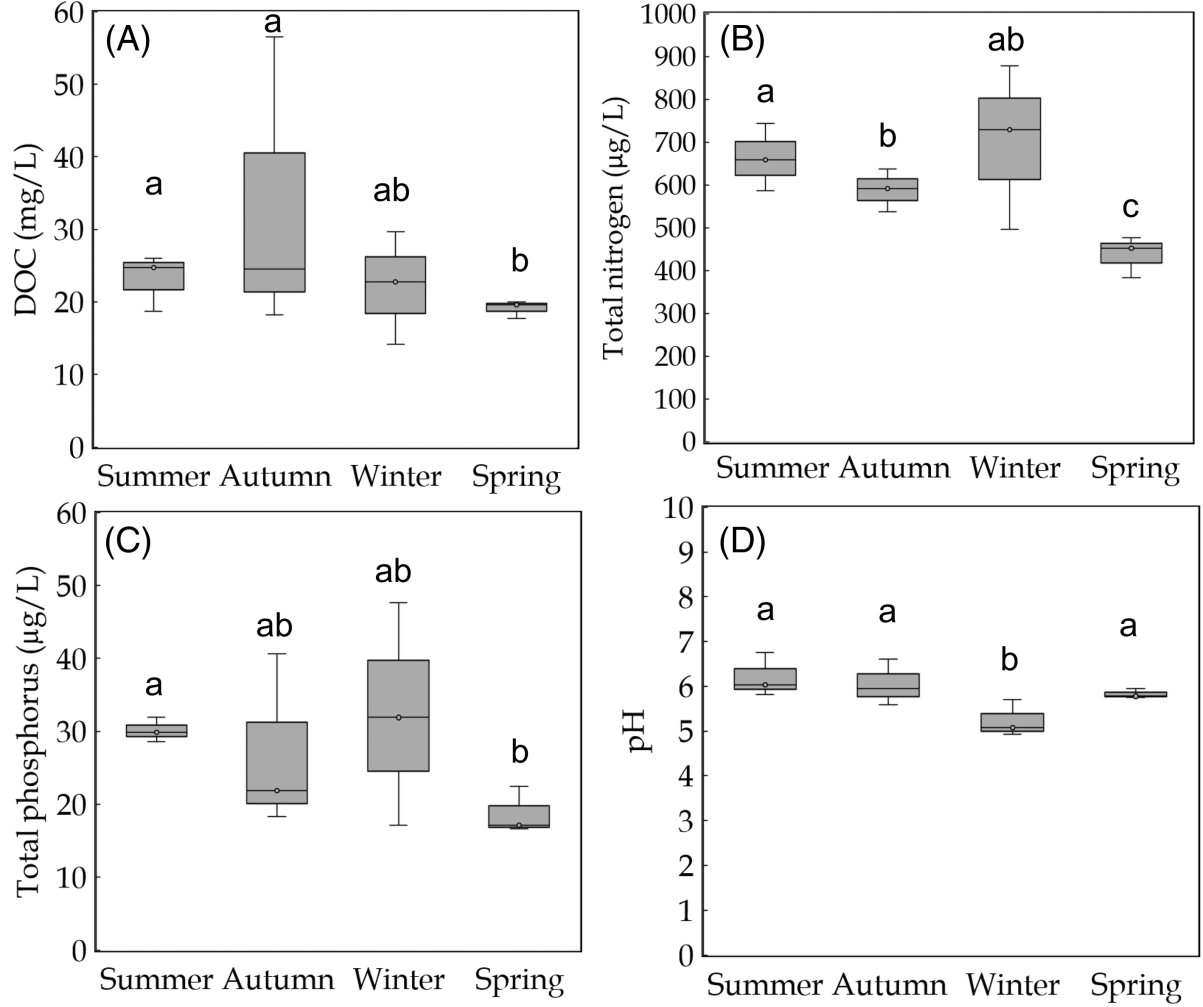
Seasonal variation in lake parameter which were shown to differ seasonally, including (A) dissolved organic carbon (DOC) concentration, (B) total nitrogen concentration, (C) total phosphorus concentration, and (D) pH. Statistical significances are marked as letters above boxes; same letter between two seasons indicates there is no difference between them, whereas different letters indicate significant difference (*p* < 0.05).

Lake water chemistry was monitored as DOC concentration, DIC concentration, TN concentration, TP concentration, and pH (Table S1). DOC content was significantly higher in summer and autumn than in spring but did not differ between other seasons (Figure 1A). DIC content did not differ seasonally. Nitrogen concentration was significantly lower in spring than in summer and winter and was also significantly lower in autumn than in summer (Figure 1B). Phosphorus concentration was significantly lower in spring than in summer but did not vary between other seasons (Figure 1C). In winter, the pH was significantly lower than in any other season (Figure 1D). Statistical results concerning biogeochemical differences between seasons are presented in Supplementary materials (Table S2).

### Microbial respiration and biomass

**FIGURE 2:**
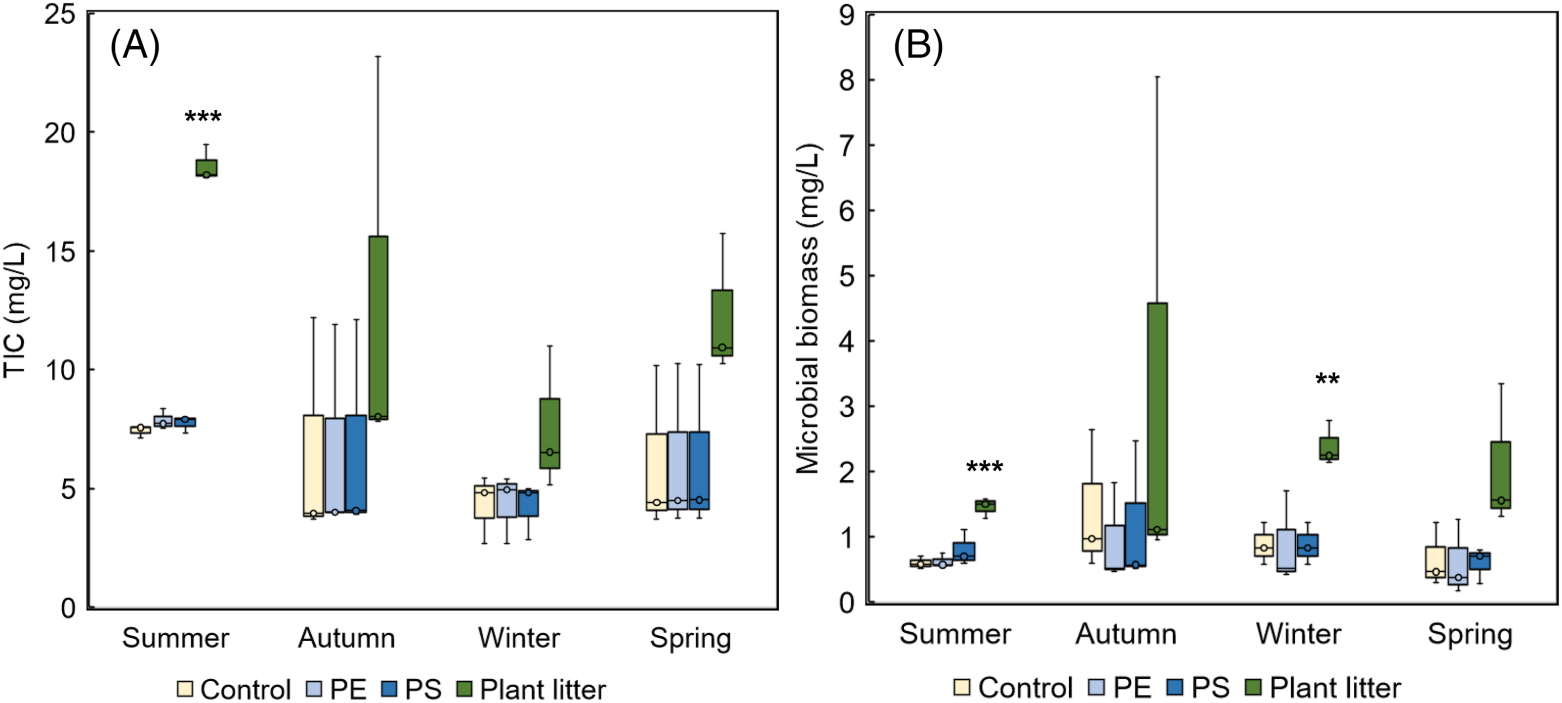
(A, B) Microbial respiration measured as total inorganic carbon (TIC) and (B) microbial biomass after the 4 weeks incubation period. Plant litter addition increased TIC and microbial biomass in comparison to control groups, whereas neither PE nor PS affected TIC or biomass. *p < 0.05, **p < 0.01, ***p < 0.001.

Microplastic additions did not enhance microbial respiration or biomass (Figure 2A,B). In contrast, plant litter addition significantly increased respiration in summer and biomass in comparison to control in summer, winter, and spring (Table S3). Moreover, control treatments revealed the level of natural variation in the TIC concentration and amount of biomass. TIC was significantly higher in summer than in winter (*p* = 0.027, *t* = 3.70), whereas biomass did not differ significantly between the two seasons (Table S4).

### Decomposition process, the biochemical fate of carbon and BGE

**FIGURE 3:**
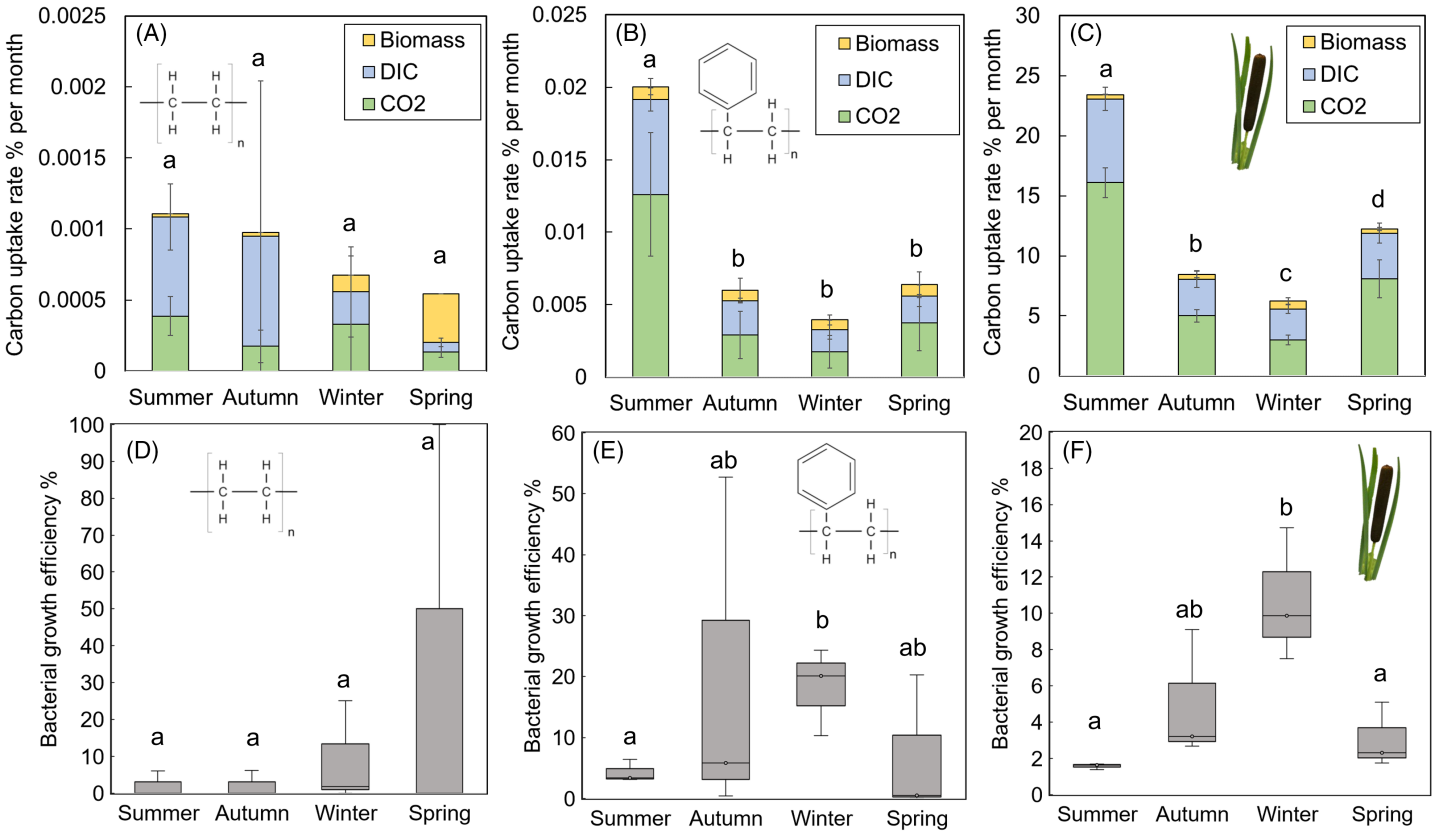
Seasonal changes in decomposition time and biochemical fates (microbial biomass, dissolved inorganic carbon [DIC], carbon dioxide [CO_2_]) of (A) polyethylene, (B) polystyrene, and (C) plant litter carbon. Seasonal variation in the utilization of substrate carbon for microbial growth was examined by determining bacterial growth efficiency (BGE) in lake waters with (D) polyethylene, (E) polystyrene, and (F) plant litter addition in each season.

The uptaken carbon from each substrate was mostly mineralized, whereas only a small proportion was bound into microbial biomass, except in spring PE treatments (Figure 3A–C). PS was mineralized significantly faster in summer, whereas the mineralization rate did not differ between other seasons (Table S5). However, although the mineralization played the most important role as a major fate of uptaken carbon, BGE was shown to vary between seasons in lake waters with PS and plant litter addition, but not with PE (Figure 3D–F). BGE indicated that PS and plant litter are used more efficiently for biomass formation in winter than in summer (*p* = 0.04, *t* = 3.24 and *p* = 0.02, *t* = 4.28, respectively). Notably, the assimilation rate of substrate carbon per se did not differ between seasons (*p* > 0.05), and differences in BGE are caused by a decrease in mineralization rates in lower temperatures.

**FIGURE 4:**
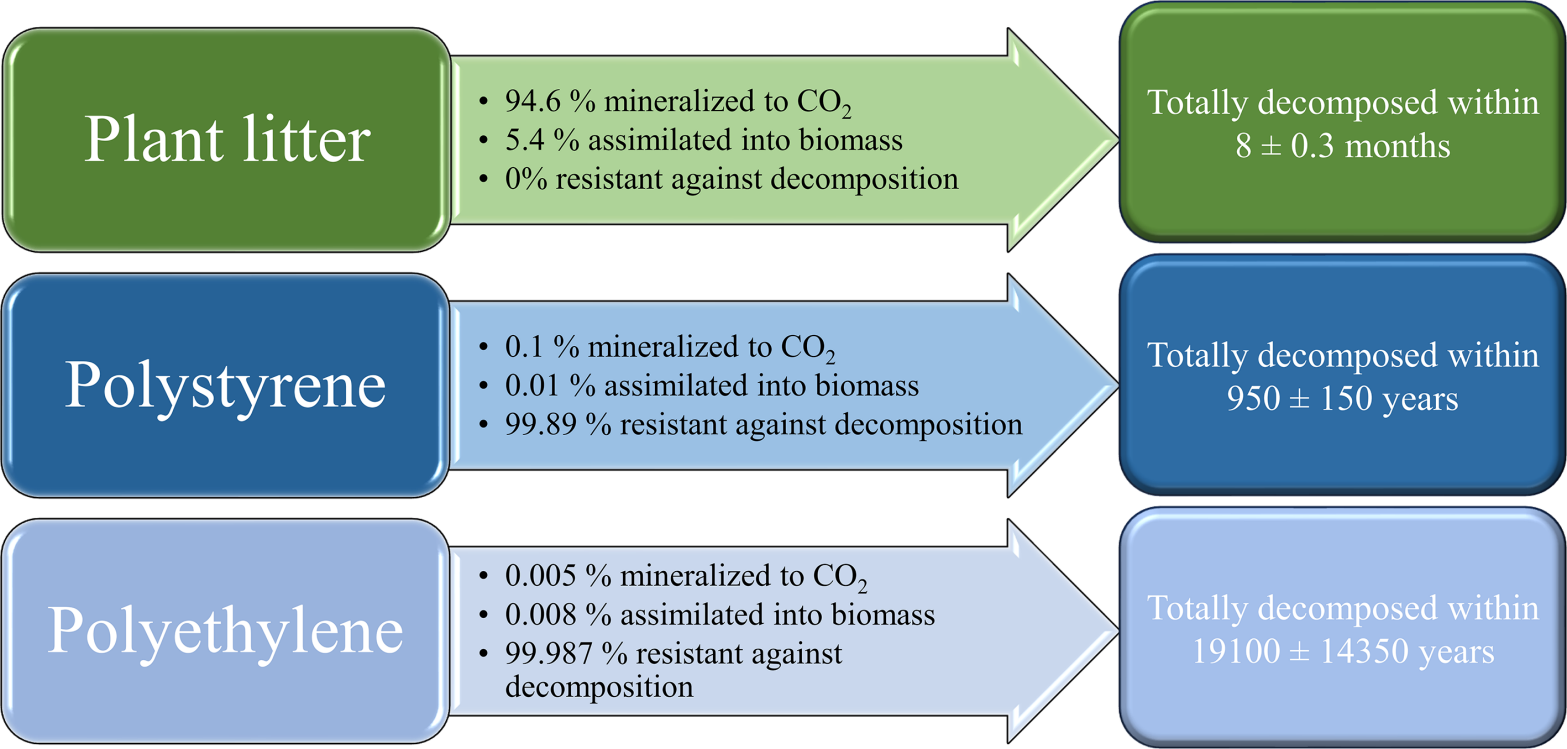
The percentual fate of carbon from each studied substrate and their resistance against decomposition (percentage of not degraded substrate) after 1 year of degradation (arrows) and the calculated total decomposition time (right squares).

PE showed high resistance against biological degradation and did not vary seasonally (*p* > 0.05). Its annual decomposition rate was only 0.007 ± 0.004% per year (Figures 3A and 4), the total decomposition rate of PE being within 19,100 ± 14,350 years. In contrast, PS was decomposed faster, reaching the annual decomposition rate of 0.11 ± 0.02% per year (Figures 3B and 4) and therein means that PS would be fully decomposed within 950 ± 150 years. Unlike with PE, PS was degraded faster in summer than in any other season, showing seasonal variation (Figure 3A–F; Table S5). The fastest degradation rate was observed in summer and was fivefold in contrast to the degradation rate during the winter season. Plant litter decomposition showed a similar fashion: decomposition rates varied between each season (Table S5), and the fastest decomposition rate was observed in summer, whereas the rate in winter was fourfold slower. The total decomposition rate of plant litter was calculated to be 8 ± 0.3 months. However, in contrast to microplastics, plant litter was efficiently utilized and even 12.6 ± 0.5% of its mass was lost per month (Figures 3C and 4).

### Compound‐specific isotope analysis of PLFAs

**FIGURE 5:**
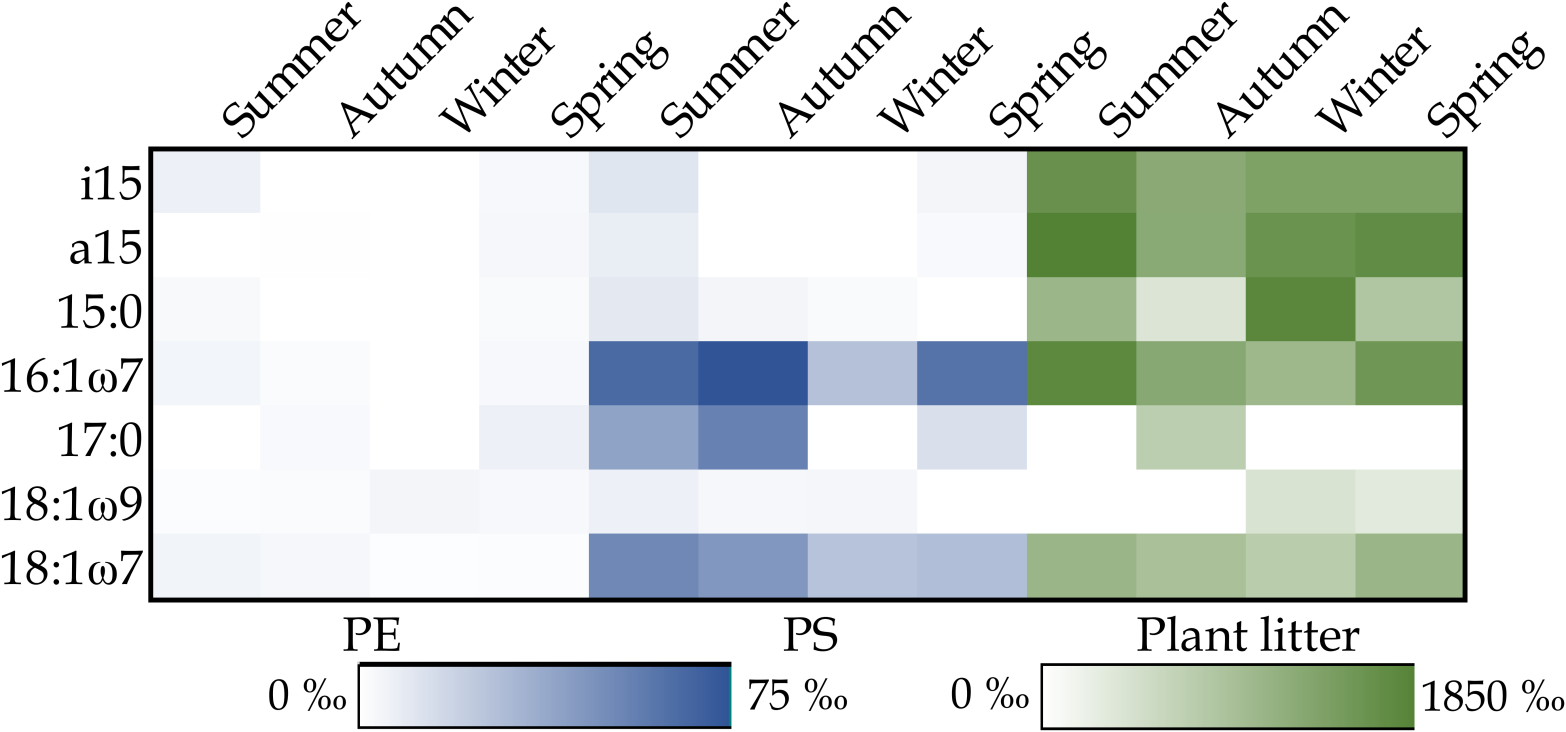
Heatmap indicating the average δ^13^C‐enrichment of specific PLFAs in PE, PS, and plant litter treatments in each season, calculated as a difference between δ^13^C‐values of control PLFAs and δ^13^C‐values of PLFAs in each substrate treatment. ^13^C‐enrichment of PLFAs differed between PE, PS, and plant litter treatments. Within a given substrate treatment, ^13^C‐enrichment of PLFAs was relatively similar between seasons.

CSIA of PLFAs was used to track the transfer of ^13^C‐substrate carbon into specific PLFAs, that can be used as biomarkers to identify active decomposers. Low ^13^C‐enrichment of PLFAs in PE treatments suggests that PE is poorly degraded (Figure 5). PLFAs i15, 16:1ω7, 18:1ω7, and 18:1ω9 had significantly higher ^13^C‐enrichment in comparison to control in summer. In winter, PLFAs a15, 16:1ω7, and 18:1ω9 showed higher ^13^C‐enrichment. In spring, ^13^C‐enrichment of PLFAs i15, a15, 15:0, 16:1ω7, and 17:0 differed significantly from control. Notably, although significant differences were found, the ^13^C‐enrichment of these PLFAs was extremely low (Figure 5).

In the PS treatment, PLFAs 16:1ω7, 18:1ω7, and 17:0 showed higher enrichment of ^13^C in comparison to other PLFAs throughout all seasons (Figure 5). Among these, PLFAs 16:1ω7 and 18:1ω7 were more ^13^C‐enriched in the PS treatments than in the control in summer, winter, and spring (Table S6). δ^13^C‐values of PLFA 17:0 strongly varied, and thus the difference in comparison to the control was not significant in the PS treatments in any season (Table S6). PLFA i15 was significantly more ^13^C‐enriched in the PS treatments in summer, winter, and spring in comparison to control waters, but the enrichment was lower than in PLFAs 17:0, 16:1ω7, and 18:1ω7 (Figure 5, Table S6).

Several PLFAs in lake waters with plant litter addition were highly ^13^C‐enriched in comparison to the control (Figure 5, Table S6). Across all seasons, the highest ^13^C‐enrichment was observed for PLFAs 16:1ω7 and 18:1ω7 and branched PLFAs i15 and a15. Additionally, PLFA 18:1ω9 was enriched with ^13^C in all seasons, but enrichment was relatively low in comparison to other PLFAs. PLFA 15:0 was relatively strongly enriched with ^13^C in summer and winter, and PLFA 17:0 was enriched with ^13^C in autumn. Fungal biomarker PLFAs 18:2ω6 and 18:3ω3 were not enriched with ^13^C in any of the treatments.

### Microbial decomposer communities

**FIGURE 6:**
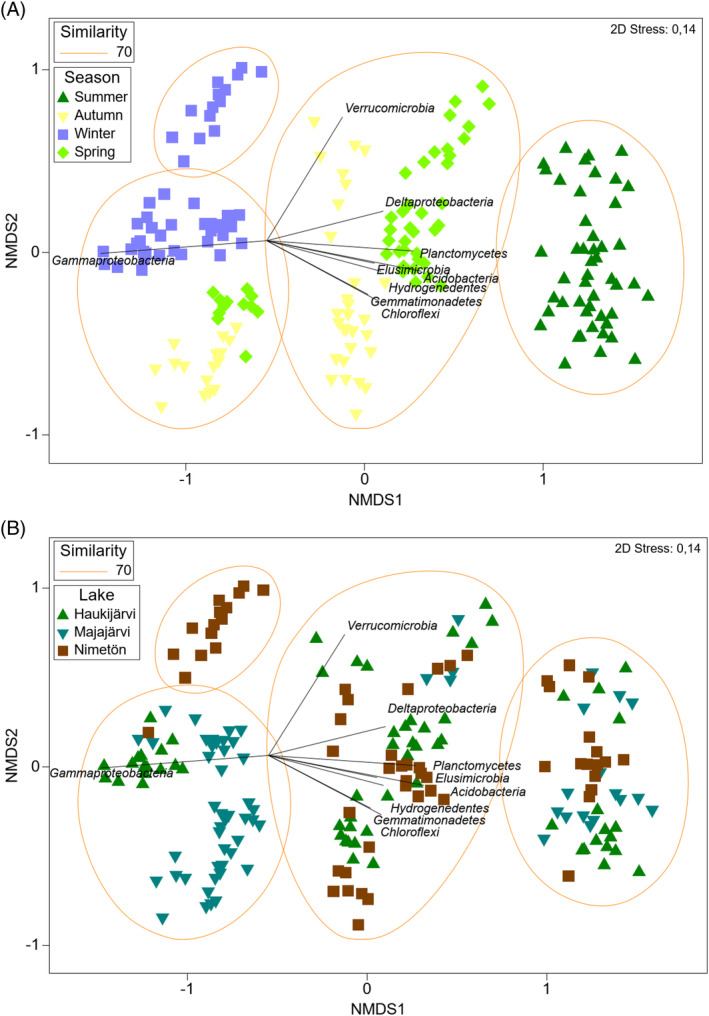
NMDS plot showing microbial classes explaining the differences between seasons, lake waters, and treatments, visualized based on (A) season and (B) lakes.

**FIGURE 7:**
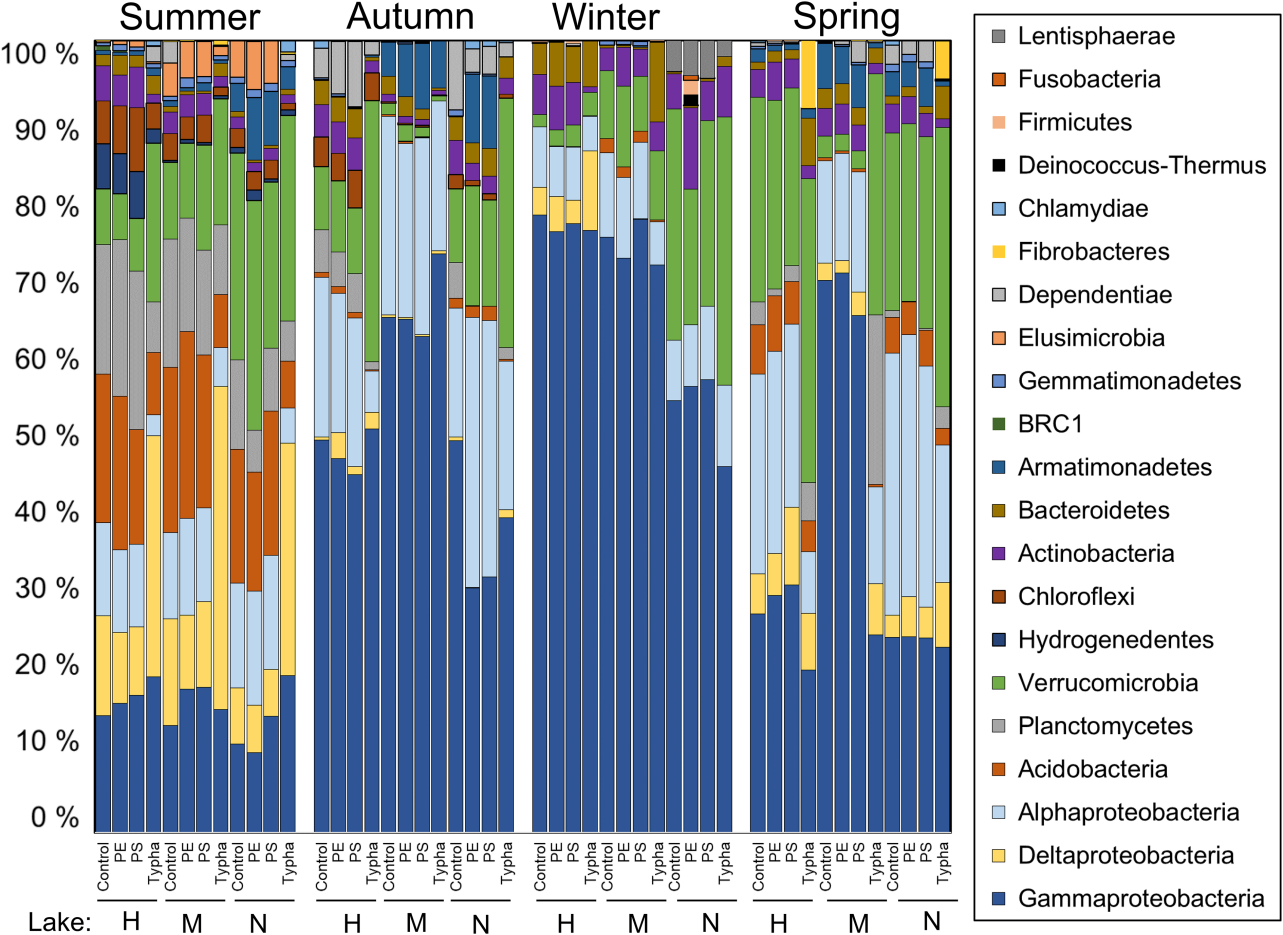
Bacterial community structures in each treatment and season at the class level, shown as relative abundances (>0.5% of all 16S rRNA sequences). Lake abbreviations: H = lake Haukijärvi, M = lake Majajärvi, N = lake Nimetön.

Microbial community structures differed significantly between seasons and lakes at the genus and the class levels (Tables S7 and S8). Communities of three lake waters were more similar within each season than within their origin lakes (Figure 6A,B). Particularly summer lake water community structures diverged from those of other seasons, which were mainly explained by the lower relative abundance of Gammaproteobacteria, whereas Acidobacteria, Planctomycetes, and Deltaproteobacteria had the highest proportions (Figures 7 and S1).

**FIGURE 8:**
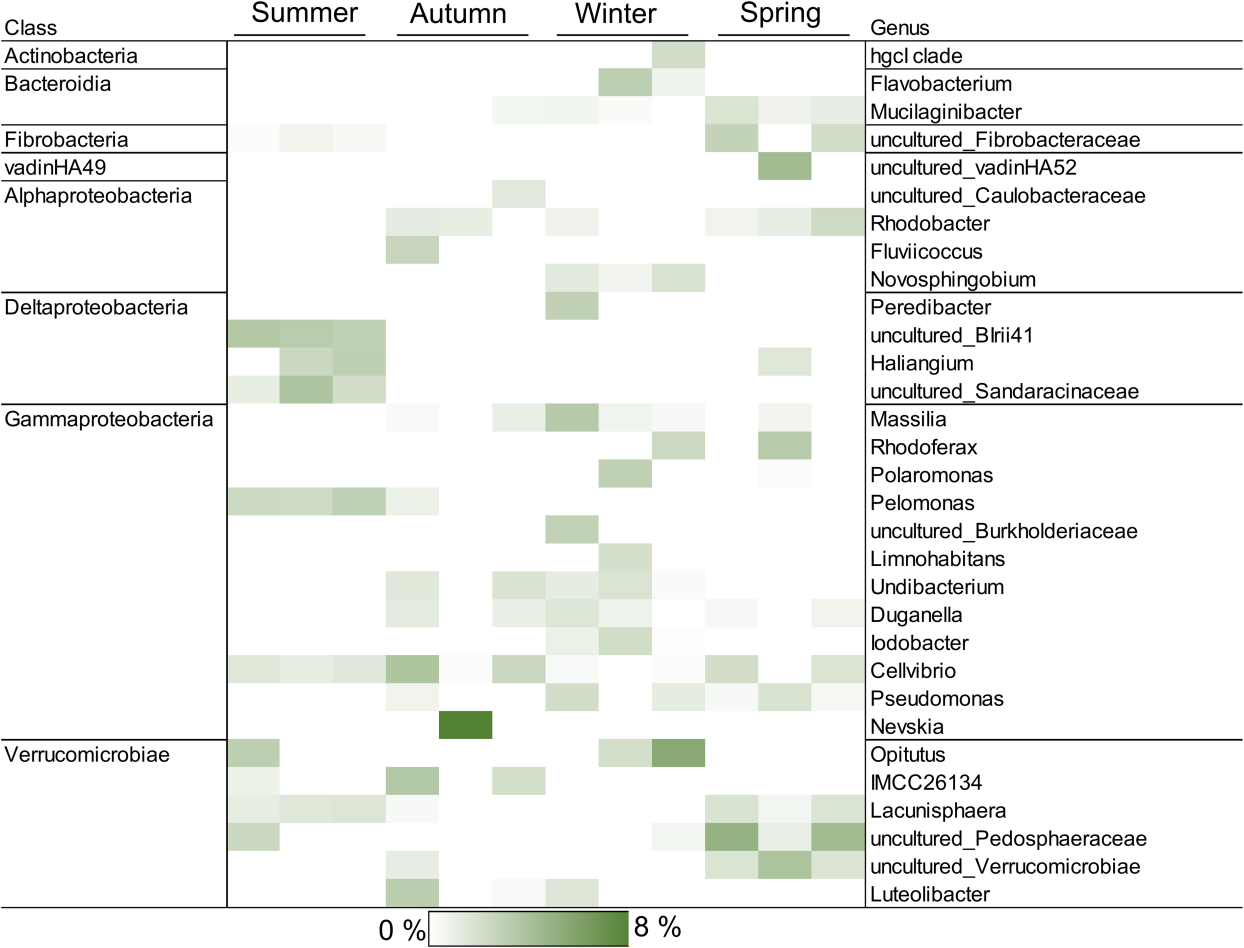
Heatmap showing bacterial genera that correlated (Spearman >0.6) with plant litter associated microbiome in NMDS analysis performed for each season. Darker green colour indicates a higher average relative abundance of a given genus, thus indicating a more important role in plant litter decomposition in contrast to lighter squares. Only genera contributing >2% in at least one lake water (*n* = 3) were included in the heatmap after which data were square root transformed for better visualization.

Plant litter addition changed community composition in each season significantly (*p* = 0.002, *t* = 2.41), and thus responses in microbial community were used to identify plant litter decomposer taxa (Figure 8). SIMPER analysis showed that changes were driven by Deltaproteobacteria (*Peredibacter* sp., uncultured Blrii41, and *Haliangium* sp.), Verrucomicrobia (e.g., *Opitutus* sp. and *Lacunisphaera* sp.), and Gammaproteobacteria (*Pelomonas* sp. and *Cellvibrio* sp.) in summer, whereas in autumn, only Verrucomicrobia (e.g., *Luteolibacter* sp.and *Opitutus* sp.) were more abundant in comparison to control (Figure 8, Table S9). Verrucomicrobia (primarily *Opitutus* sp.), Bacteroidetes (*Flavobacterium* sp. and *Mucilaginibacter* sp.), and Deltaproteobacteria (*Peredibacter* sp.) increased their abundance in winter lake waters with plant litter addition. In spring, vadinHA49, Verrucomicrobia (*Lacunisphaera* sp. and two uncultured genera from Pedoshaeraceae and Verrucomicrobiae), Fibrobacteria, and Deltaproteobacteria (*Haliangium* sp.) were shown to be typical to lake waters with plant litter addition (Figure 8).

**FIGURE 9:**
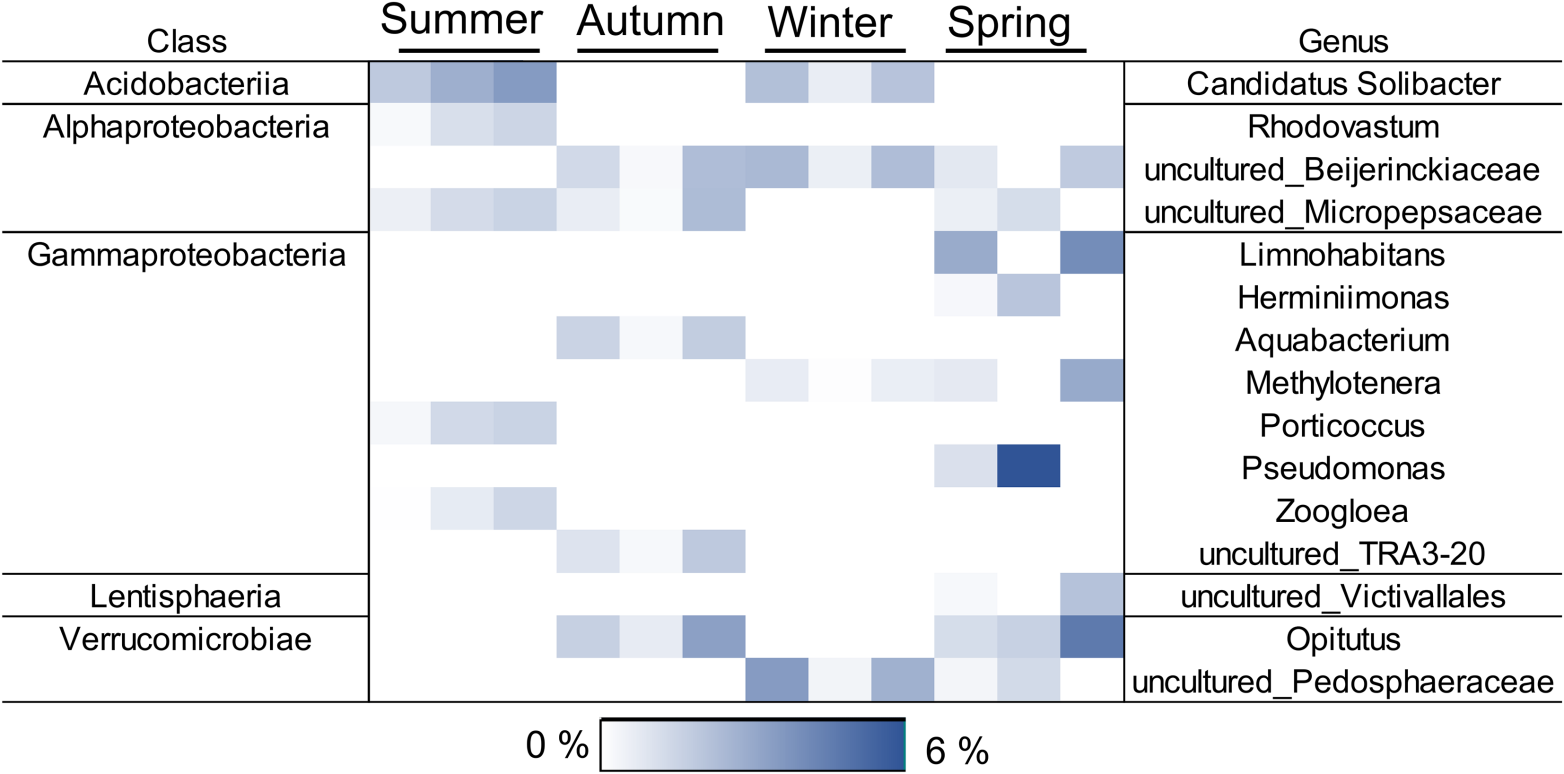
Heatmap showing bacterial genera that correlated with the fast PS decomposition rate in NMDS analysis performed for each season. Darker blue colour indicates a higher average relative abundance of a given genus, thus indicating a more important role in PS decomposition in contrast to lighter squares. Only genera contributing >2% in at least one lake water (*n* = 3) were included in the heatmap after which data were square root transformed for better visualization.

Plastic additions did not affect microbial community structures (*p* > 0.05). Since PE was extremely slowly decomposed, it was meaningless to identify the decomposer taxa. In contrast, PS was decomposed in all seasons. Lake‐specific differences in the decomposition rates allowed the identification of microbial taxa correlating towards a faster degradation rate within each season (Figure S2). Taxa that correlated towards lake water microbiome with the highest decomposition rate within each season and which correlated with the decomposition rate itself were listed (Tables S10–S14). As a total, 15 potential genera with relative abundance >2% were identified as the most potential PS decomposers (Figure 9). These genera belonged to classes Acidobacteriia (Candidatus *Solibacter* sp.), Alpha‐ and Gammaproteobacteria (e.g., *Rhodovastum* sp. *Limnohabitans* sp., *Porticoccus* sp. and *Pseudomonas* sp.), Lentisophaeria (uncultured Victivallales), and Verrucomicrobia (*Opitutus* sp. and uncultured Pedosphaeraceae).

## DISCUSSION

### Seasonal variation in decomposition rates of microplastics and plant litter

PE was extremely slowly degraded in all seasons, and its decomposition rate did not vary seasonally. The PE degradation rate was much lower than previously measured (Taipale et al., 2019, 2022). Although we collected water from the same lakes as in the studies mentioned above and even used the same experimental temperature in summer as Taipale et al. (2022), DOC levels were higher than what has been reported previously (Taipale et al., 2019). Thus, there seems to be annual variation in water chemistry and potentially also in nutrient content and microbial community structures which may affect the PE decomposition rate (DeGasparro et al., 2020; Singh et al., 2014; Yindong et al., 2021). Another explanation for differing decomposition rates is the lack of photooxidation that commonly initializes polymer degradation, further accelerating physical degradation mechanisms and biological degradation (Ali et al., 2021; Chamas et al., 2020; Priya et al., 2022). In contrast, PS was decomposed at the fastest rate in summer (fourfold in contrast to winter) and relatively constantly during all other seasons, although variation between lake waters was observed as well. In addition to the lack of photooxidation, the huge difference in decomposition rates of the two microplastics is likely explained by the chemical structure: since microbes can hydrolyse heteroatomic and aromatic microplastics more efficiently (Brunner et al., 2018; Debroas et al., 2017; Vesamäki et al., 2022), aromatic PS is more prone to degradation than aliphatic PE. By using similar methods than in the present study, we have previously estimated that PS would degrade within 500 ± 150 years in humic lakes (Vesamäki et al., 2022). Thus, the decomposition time of PS is twice as long (950 ± 150 years) as what has been previously estimated, when seasonal variation is taken into account. Additionally, the stimulating effect of UV radiation on plastic degradation (Chamas et al., 2020) and thermo‐oxidation (Anderson et al., 2016) were not considered in the current study, and thus the microplastic degradation may be faster in natural conditions. On the other hand, the slow decomposition of plastics in lower temperatures suggests that their permanence in fragile arctic and subarctic ecosystems, where microplastic pollution has been observed (Bergmann et al., 2022; Citterich et al., 2023), is even higher than in southern ecosystems.

Unlike among studied microplastics, the decomposition rate of plant litter varied between each season. The fastest decomposition of plant litter was detected in summer, when the decomposition was fourfold higher in contrast to winter, corresponding to what has been found in a previous study (van Dokkum et al., 2002). Fast utilization of plant litter supports a current view that terrestrial OM sources boost microbial activity by offering a new source of elements and energy (Attermeyer et al., 2013; Brett et al., 2017). Notably, the decomposition of both PS and plant litter continued also in winter, showing that microbes are capable of cleaving not only labile but recalcitrant polymer bonds as well in low temperatures. Thus, degradation processes are not limited only to growth season.

Although temperature explained most of the variation in decomposition rates, the nonlinear development of the decomposition rate of PS and plant litter across temperature gradient (as seen as relatively slow decomposition of plant litter and PS in spring in contrast to summer) suggests that other environmental parameters affected decomposition as well. These could include changes in the availability of carbon sources, for example, via soil OM leaching from surrounding terrestrial ecosystem within thawing snow (Kim et al., 2017; Wipf et al., 2015) or from leaf input in autumn (Attermeyer et al., 2013; Sebetich & Horner‐Neufeld, 2000; Singh et al., 2014) or lake's changing nutrition content due to external and/or internal loadings (Wang et al., 2019; Yindong et al., 2021). Indeed, concentrations of dissolved nitrogen and phosphorus were lower in spring than in summer. Higher nutrient concentration may accelerate decomposition processes (Carpenter & Adams, 1979; DeGasparro et al., 2020; Grasset et al., 2017), and it is known that microplastics can absorb nutrients from the surrounding environment and create favourable conditions for microbes (Du et al., 2022; Shen et al., 2019; Yang et al., 2020). Therein, in addition to lower temperature, nutrient content is a likely cause for a relatively high difference in decomposition rates between spring and summer and explains the nonlinear changes in decomposition rates across seasons. In addition, DOC content which has previously been reported to positively correlate with plastic degradation rate (Taipale et al., 2019) was higher in autumn than in spring, but the decomposition rate of plant litter was higher in spring and PS decomposition rate was equal between these two seasons. Thus, environmental factors affecting seasonal decomposition processes are primarily temperature and secondarily nutrient concentration, whereas DOC concentration was not found to affect the decomposition among humic lakes, although DOC content may be at least an indicative factor for decomposition rate among wider DOC gradient (Taipale et al., 2019; Vesamäki et al., 2022). Since we sampled lakes only once per season, the accuracy of decomposition rates at the annual level could be improved by examining more closely the within‐season variation of decomposition rates.

### Seasonal variation in the biochemical fate of carbon and BGE

Our results show that carbon from plant litter and petroleum‐based plastics can be released back into the atmosphere via microbial decomposition whereas a smaller proportion is assimilated into microbial biomass and further can be integrated into the aquatic food web. Since lake nutrient contents were at a similar level in summer and in winter, the fate of carbon seems to be determined mainly by the temperature that regulates mineralization (Gudasz et al., 2010; Hall et al., 2008). However, our results showed that the faster mineralization rate in summer was not only caused by higher microbial respiration, but also Δδ^13^C values were higher in summer than in winter. This indicates that higher temperature accelerates PS biodegradation not only by promoting microbial respiration but also by the efficiency of degradation pathways per se.

On average, 89 and 95% of uptaken carbon from PS and plant litter, respectively, was mineralized to CO_2_ and therein utilized as an energy source. The mineralization rate of plant litter and PS varied between seasons and was the fastest in the summer. In contrast, the remaining proportions of uptaken carbon from PS and plant litter were assimilated into biomass at an equal rate between all seasons (11 and 5%, respectively), thus offering a stable subsidy to the aquatic food web throughout seasons. A higher proportion of mineralized microplastic carbon in relation to assimilation has been observed also for instance in mealworm's gut microbiota (Yang et al., 2015) and a fungal culture (Rohrbach et al., 2024). In contrast, allochthonous carbon is used mostly for microbial growth in freshwater lakes (Taipale et al., 2023; Vesamäki et al., 2022), contradicting the findings of the present study. However, different results between studies may be explained by differing incubation periods; longer incubation time likely increases microbial biomass, and if Δδ^13^C values of biomass remain at a similar level, the assimilation: mineralization ratio would likely increase as well. This seems likely from the point of view of PS and plant litter mineralization, which are known to decrease or remain at a similar level after 3 weeks of incubation (Vesamäki et al., 2022), favouring biomass as the endpoint of carbon at later stages of decomposition. To confirm this, experimental succession‐dependent carbon cycling would be needed. In addition, the fate of carbon may differ between different lake types (Taipale et al., 2022; Vesamäki et al., 2022). Overall, the fate of carbon seems to be affected by both incubation time, seasonal changes, and environmental conditions.

### Identification of plant litter and microplastic decomposers

As observed by other studies (Duarte et al., 2016; Lew et al., 2015), microbial community compositions in studied lakes varied seasonally. Moreover, our results suggest that several microbial taxa are capable of utilizing plant litter as a carbon and energy source in every season. In comparison to control waters, 31 genera were shown to increase their abundance after plant litter addition, suggesting that these taxa participate in terrestrial litter carbon cycling in humic lakes. Recognized genera belonged to Actinobacteria, Bacteroidia, Fibrobacteria, vadinHA49, Proteobacteria (in summer, particularly Deltaproteobacteria), and Verrucomicrobia, supporting previous studies of leaf litter decomposition (Vesamäki et al., 2022; Zhao et al., 2021). Particularly Verrucomicrobia was found to enrich in several seasons, suggesting their importance in plant litter carbon uptake. Since Verrucomicrobia are commonly recognized as polysaccharide utilizers (Cardman et al., 2014; Sichert et al., 2020), and they respond to the elevated nutrient availability (Lindström et al., 2004; Parveen et al., 2013; Tran et al., 2018) offered by the plant litter input, it seems likely that they utilized plant litter polysaccharides and low molecular weight compounds as their carbon source in the present study. Bacteria may benefit also the particulate form of plant litter: Deltaproteobacteria Verrucomicrobia has been reported to be particle‐associated (Morrison et al., 2017; Parveen et al., 2013).

In later phases of the decomposition process, microbial succession may favour more specific taxa that are capable of utilizing recalcitrant polymers (Bonanomi et al., 2019; Newman et al., 2015; Zhao et al., 2021). This includes fungi. However, after our one‐month incubation period, PLFA 18:1ω9 was the only fungal biomarker PLFA which was found to enrich with ^13^C. PLFA 18:1ω9 is characteristic of both fungi and Planctomycetes (Elshahed et al., 2007; Willers et al., 2015). The dual role of PLFA 18:1ω9 as a biomarker challenges the interpretation of results, and the use of PLFA 18:1ω9 as a biomarker should be carried out with proper caution. The strongest enrichment with ^13^C in PLFA 18:1ω9 was found in plant litter treatments in spring and winter. In spring, the increased relative abundance of Planctomycetes in plant litter treatments suggests that the signal originates from Planctomycetes rather than fungi, supporting previous studies (Taipale et al., 2022; Vesamäki et al., 2022). In contrast, Planctomycetes were low in abundance in winter and therein less likely to participate in the decomposition process of plant litter. It is more likely that fungi participated in the decomposition in winter, although other fungal biomarkers (Willers et al., 2015) were not enriched with ^13^C. Since we did not analyse fungal community structures, potential fungal decomposers cannot be determined based on our data. In other seasons and treatments, the low enrichment of PLFA 18:1ω9 and other fungal biomarkers with ^13^C suggests that bacteria (other than Planctomycetes) were driving the decomposition processes. Overall, the CSIA of PLFAs supported the conclusion that the decomposition of plant litter was driven by several bacterial classes in each season and was in line with the results of microbial community analysis and previous studies (Hayer et al., 2016, 2022; Purahong et al., 2016; Taipale et al., 2023; Vesamäki et al., 2022).

Our results suggest that PS decomposer community composition varies seasonally at the genus level, but not at the class level. Overall, 15 genera, belonging to Acidobacteria, Alpha‐ and Gammaproteobacteria, Lentisphaerae, and Verrucomicrobia correlated towards fast decomposition rate in different seasons. Since the relative abundance alone does not indicate the degradation, the CSIA of PLFAs was combined with microbial community analysis to exclude unlikely PS decomposers. The commonly found major PLFA in Verrucomicrobia is a15 (Op den Camp et al., 2009), which became only weakly ^13^C‐enriched, suggesting that Verrucomicrobia were not driving degradation strongly but may have contributed. In contrast, high ^13^C‐enrichment was observed for PLFAs of 16:1ω7 and 18:1ω7, typical to gram‐negative Proteobacteria and Acidobacteria (Willers et al., 2015), supporting previous studies (Taipale et al., 2019, 2022, 2023; Vesamäki et al., 2022). Among Acidobacteria, Candidatus *Solibacter* sp. was the only representative that was associated with PS carbon utilization. However, Candidatus *Solibacter* sp. seems unlikely to decompose PS, since it uses labile carbohydrates as its main carbon sources (Kulichevskaya et al., 2010; Rawat et al., 2012), and its abundance is inhibited by high doses of PS in soils (Qin et al., 2023). Thus, results suggest that PS decomposers belong to Proteobacteria. Particularly Alpha‐ and Gammaproteobacteria are potential PS decomposers whereas Deltaproteobacteria are unlikely to initialize the decomposition process since our analysis showed that the members of Deltaproteobacteria did not correlate towards fast PS decomposition in any season. The importance of Alpha‐ and Gammaproteobacteria in the initialization of the PS decomposition was supported by the CSIA analysis which indicated that the same microbial phyla drive the decomposition of PS in studied lakes in each season. Three of the identified gammaproteobacterial genera (*Limnohabitans* sp., *Herminiimonas* sp., and *Aquabacterium* sp.) belong to the family Burkholderiaceae, whose members have been recognized to degrade aromatic polymers (Pérez‐Pantoja et al., 2012), supporting the likelihood that they participate to PS decomposition process in humic lakes. An uncultured member from the family Beijerinckiaceae (Alphaproteobacteria) whose members have been found in the plastisphere (Nguyen et al., 2021), correlated with faster decomposition in autumn, suggesting its strong association with PS and potential capacity to utilize PS carbon. Overall, our results support previous findings that Alpha‐ and Gammaproteobacteria involve numerous promising microplastic degrader candidates (Roager & Sonnenschein, 2019; Sekiguchi et al., 2011). These two classes seem to play an important role as plastic degraders in lake ecosystems throughout the year.

### Conclusions

Our study with two plastics and plant litter showed that the decomposition of plastics and plant litter continued over seasons. Microbes were shown to regulate the cycling of microplastic and OM carbon depending on seasonal parameters, particularly temperature, which affected the microbial respiration and the mineralization of substrate carbon. Our results highlight the ability of freshwater microbiome to use both recalcitrant and labile carbon sources throughout the year, even at low temperatures. PE showed a much lower rate of decomposition than previously measured in humic lakes, potentially due to lack of abiotic initiation, for example, by UV‐radiation which was not taken into account here. In contrast, provided PS was decomposed in all seasons. PS was degraded at the fastest rate in summer and relatively constantly during all other seasons. In a similar fashion, plant litter decomposition rates varied seasonally. Its decomposition supported the formation of microbial biomass in all seasons, showing that biodegradation of fallen leaves is important support for microbes throughout seasons. Plant litter was degraded by a wider range of microbes than microplastics. Additionally, microbial decomposers of plant litter and plastics varied seasonally. Our results show how the decomposition of labile OM and extremely recalcitrant microplastics continues over seasons, revealing the never‐ending process of microbial decomposition. Our study emphasizes the importance of seasonal variation in microbial processes, polymer degradation, and carbon cycling. This variation should be taken into account more often in the field of environmental microbiology.

## AUTHOR CONTRIBUTIONS

**Jussi S. Vesamäki:** Conceptualization (equal); data curation (lead); formal analysis (equal); investigation (lead); project administration (equal); visualization (lead); writing – original draft (lead); writing – review and editing (lead). **Miikka B. Laine:** Investigation (supporting); writing – review and editing (supporting). **Riitta Nissinen:** Conceptualization (equal); resources (supporting); supervision (equal); writing – review and editing (supporting). **Sami J. Taipale:** Conceptualization (equal); funding acquisition (lead); project administration (lead); resources (lead); supervision (equal); writing – review and editing (supporting).

## CONFLICT OF INTEREST STATEMENT

The authors declare no conflicts of interest.

## Supporting information


**DATA S1** Supplementary Information.

## Data Availability

All raw sequence reads are available in the NCBI database under BioProjects PRJNA1020678 (https://www.ncbi.nlm.nih.gov/bioproject/1020678) and PRJNA1020740 (https://www.ncbi.nlm.nih.gov/bioproject/1020740).
