# Supplementary material for: Plastic and terrestrial organic matter degradation by the humic lake microbiome continues throughout the seasons
Source: Environ Microbiol Rep. 2024 Jun 9;16(3):e13302. doi: 10.1111/1758-2229.13302 (PMC11162827; doi:10.1111/1758-2229.13302)
Supplement: Supplementary file 1 — DATA S1 Supplementary Information. [file EMI4-16-e13302-s001.docx]

Supplemental material for

Plastic and terrestrial organic matter degradation by the humic lake microbiome continues over seasons

Jussi S. Vesamäki^a^, Miikka B. Laine ^a^, Riitta Nissinen^a,b^, Sami J. Taipale^a^.

^a^ Department of Biological and Environmental Science, University of Jyväskylä, P.O. Box 35 (YA), 40014, Finland

^b^Department of Biology, University of Turku, 20014 Turku Finland

**Corresponding author**: Jussi S. Vesamäki

**Email:** jussi.s.vesamaki@jyu.fi


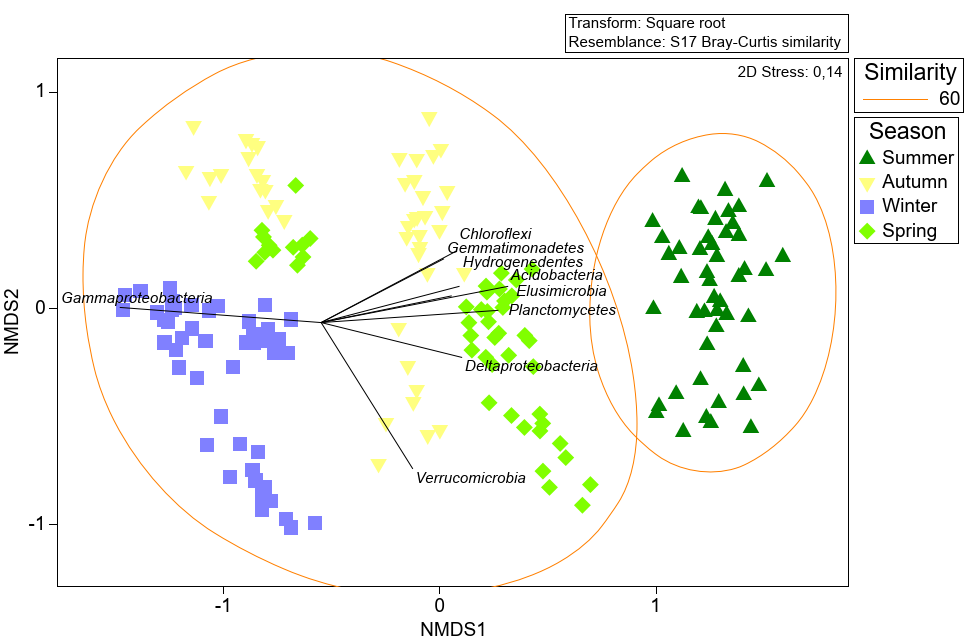


Supplemental Figure S1. NMDS showing correlations towards seasons at the class level (>0.5% of all 16S rRNA sequences).


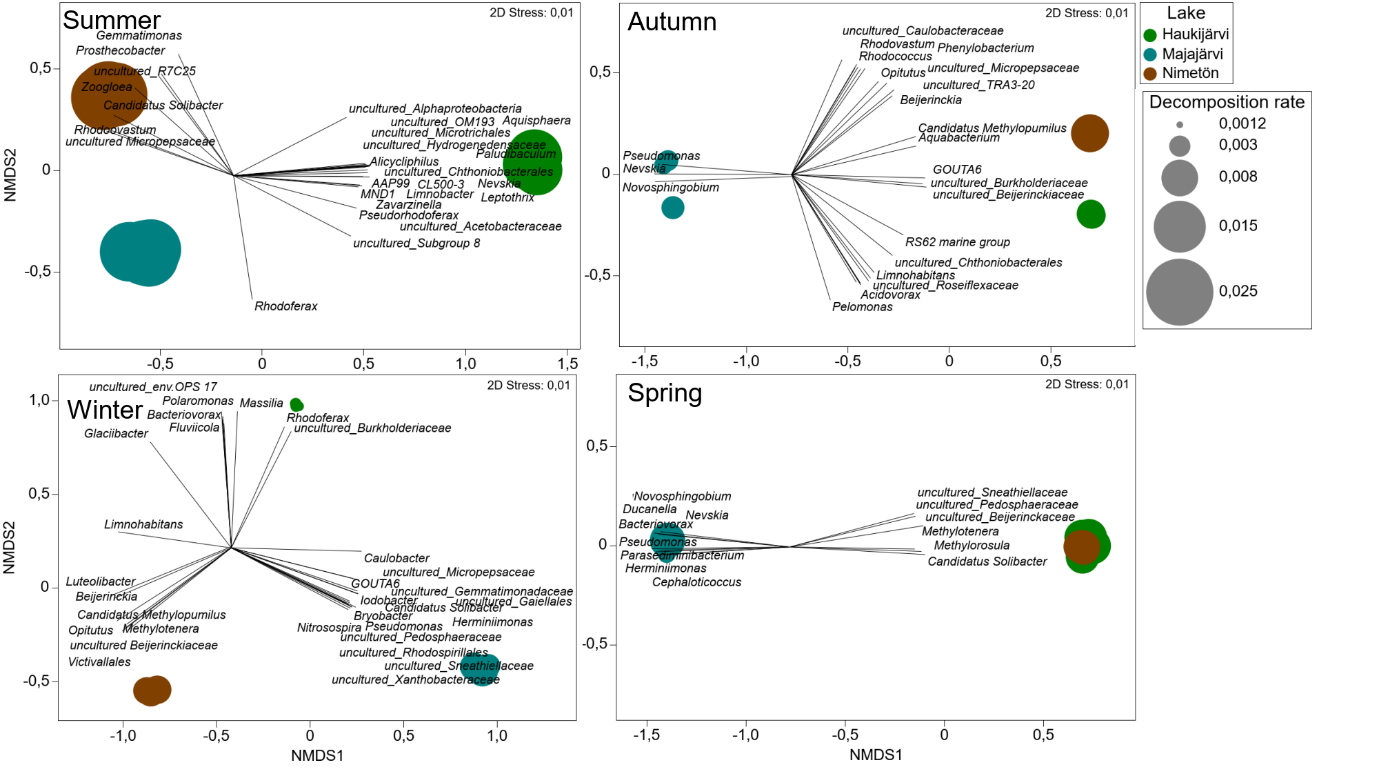


Supplemental Figure S2. NMDS analysis conducted to identify microbial genera correlating towards the fastest PS decomposition rate in each season.

Supplemental table S1. Biogeochemical parameters of studied lake waters in each season, shown as averages and standard deviations of dissolved organic carbon (DOC) concentration, dissolved inorganic carbon (DIC) concentration, total nitrogen (TN) concentration, total phosphorus (TP) concentration, and pH.

|  |  | **DOC mg/L** | **DIC mg/L** | **TN mg/L** | **P ug/L** | **pH** |
| --- | --- | --- | --- | --- | --- | --- |
| **Haukijärvi** | **Summer** | 18.7 ± 1.1 | 3.01 ± 0.19 | 0.59 ± 0.03 | 29.9 ± ND | 6.62 ± 0.27 |
|  | **Autumn** | 18.2 ± 0.7 | 1.65 ± 0.01 | 0.54 ± 0.04 | 18.3 ± ND | 6.60 ± 0.03 |
|  | **Winter** | 14.2 ± 0.2 | 2.40 ± 0.12 | 0.50 ± 0.01 | 17.1 ± 1.1 | 5.70 ± 0.20 |
|  | **Spring** | 20.0 ± 0.2 | 1.17 ± 0.02 | 0.48 ± 0.02 | 17.1 ± 4.0 | 5.78 ± 0.01 |
| **Majajärvi** | **Summer** | 24.7 ± 0.6 | 1.88 ± 0.35 | 0.66 ± 0.02 | 31.9 ± ND | 5.83 ± 0.42 |
|  | **Autumn** | 56.5 ± 0.3 | 3.11 ± 0.24 | 0.59 ± 0.01 | 21.9 ± ND | 5.58 ± 0.08 |
|  | **Winter** | 22.8 ± 0.3 | 0.96 ± 0.04 | 0.73 ± 0.04 | 31.9 ± 4.4 | 4.93 ± 0.34 |
|  | **Spring** | 19.6 ± 0.1 | 2.53 ± 0.13 | 0.45 ± 0.01 | 22.5 ± 3.7 | 5.75 ± 0.05 |
| **Nimetön** | **Summer** | 26.0 ± 0.4 | 1.71 ± 0.09 | 0.74 ± 0.06 | 28.6 ± ND | 5.62 ± 0.39 |
|  | **Autumn** | 24.6 ± 0.4 | 1.18 ± 0.03 | 0.64 ± 0.03 | 40.6 ± ND | 5.94 ± 0.04 |
|  | **Winter** | 29.7 ± 0.5 | 1.69 ± 0.14 | 0.88 ± 0.06 | 47.6 ± 1.4 | 5.08 ± 0.05 |
|  | **Spring** | 17.7 ± 0.2 | 1.05 ± 0.03 | 0.38 ± 0.02 | 16.5 ± 0.8 | 5.95 ± 0.02 |

Supplemental table S2. Statistical test results from pairwise PERMANOVA comparing seasonal differences in dissolved organic carbon content (DOC), dissolved inorganic carbon content (DIC), total nitrogen (TN) concentration, total phosphorus (TP) content, and pH. Significant (*p* < 0.05) differences are bolded. MC=Monte Carlo’s test.

|  | Groups | P(MC) | t | Unique perms |  |
| --- | --- | --- | --- | --- | --- |
| DOC | Summer, Winter | 0.671 | 0.38792 | 909 |  |
|  | Summer, Spring | **0.003** | 3.8338 | 832 |  |
|  | Summer, Autumn | 0.133 | 1.6485 | 844 |  |
|  | Winter, Spring | 0.123 | 1.584 | 800 |  |
|  | Winter, Autumn | 0.061 | 1.9622 | 907 |  |
|  | Spring, Autumn | **0.012** | 2.7394 | 911 |  |
| DIC | Summer, Autumn | 0.448 | 0.7602 | 995 |  |
|  | Summer, Winter | 0.082 | 1.8218 | 998 |  |
|  | Summer, Spring | 0.046 | 2.1949 | 995 |  |
|  | Autumn, Winter | 0.548 | 0.63141 | 998 |  |
|  | Autumn, Spring | 0.328 | 0.97899 | 991 |  |
|  | Winter, Spring | 0.654 | 0.47167 | 997 |  |
| TN | Summer, Winter | 0.554 | 0.63082 | 916 |  |
|  | Summer, Spring | **0.001** | 8.5495 | 959 |  |
|  | Summer, Autumn | **0.022** | 2.4667 | 837 |  |
|  | Winter, Spring | **0.001** | 5.2689 | 924 |  |
|  | Winter, Autumn | 0.062 | 1.9448 | 952 |  |
|  | Spring, Autumn | **0.001** | 7.3823 | 935 |  |
| TP | Summer, Winter | 0.833 | 0.23331 | 7 |  |
|  | Summer, Spring | **0.006** | 5.3807 | 10 |  |
|  | Summer, Autumn | 0.672 | 0.45857 | 10 |  |
|  | Winter, Spring | 0.210 | 1.4953 | 7 |  |
|  | Winter, Autumn | 0.650 | 0.47047 | 10 |  |
|  | Spring, Autumn | 0.296 | 1.1444 | 10 |  |
| pH | Summer, Autumn | 0.919 | 0.1051 | 263 |  |
|  | Summer, Winter | **0.002** | 3.7215 | 471 |  |
|  | Summer, Spring | 0.240 | 1.1904 | 214 |  |
|  | Autumn, Winter | **0.003** | 4.4267 | 463 |  |
|  | Autumn, Spring | 0.128 | 1.6509 | 193 |  |
|  | Winter, Spring | **0.001** | 4.7721 | 372 |  |
|  |  |  |  |  |  |

Supplemental table S3. Statistical test results from pairwise PERMANOVA showing differences between in total inorganic carbon (TIC) content and microbial biomass in each treatment (lake water with PE, PS, or plant litter (*Typha latifolia*) addition) in comparison to control (no substrate addition) in each season.

| **Season** | **Parameter** | **Groups** | **t** | **P(MC)** | **perms** |
| --- | --- | --- | --- | --- | --- |
| **SUMMER** | **TIC** | Control, PE | 1.578 | 0.186 | 10 |
|  |  | Control, PS | 1.206 | 0.289 | 10 |
|  |  | Control, Typha | 23.903 | 0.001*** | 10 |
|  | **Biomass** | Control, PE | 0.299 | 0.764 | 10 |
|  |  | Control, PS | 1.183 | 0.287 | 10 |
|  |  | Control, Typha | 8.029 | 0.001*** | 10 |
| **AUTUMN** | **TIC** | Control, PE | 0.003 | 0.999 | 10 |
|  |  | Control, PS | 0.022 | 0.986 | 10 |
|  |  | Control, Typha | 1.104 | 0.301 | 10 |
|  | **Biomass** | Control, PE | 0.600 | 0.591 | 10 |
|  |  | Control, PS | 0.242 | 0.836 | 10 |
|  |  | Control, Typha | 0.813 | 0.482 | 10 |
| **WINTER** | **TIC** | Control, PE | 0.030 | 0.976 | 10 |
|  |  | Control, PS | 0.087 | 0.943 | 10 |
|  |  | Control, Typha | 1.674 | 0.183 | 10 |
|  | **Biomass** | Control, PE | 0.008 | 0.995 | 10 |
|  |  | Control, PS | 0.006 | 0.999 | 10 |
|  |  | Control, Typha | 5.536 | 0.004** | 10 |
| **SPRING** | **TIC** | Control, PE | 0.023 | 0.985 | 10 |
|  |  | Control, PS | 0.021 | 0.984 | 10 |
|  |  | Control, Typha | 2.323 | 0.076 | 10 |
|  | **Biomass** | Control, PE | 0.125 | 0.903 | 10 |
|  |  | Control, PS | 0.200 | 0.861 | 10 |
|  |  | Control, Typha | 2.016 | 0.111 | 10 |

Supplemental table S4. Statistical test results from pairwise PERMANOVA conducted for controls to detect natural seasonal variation in amounts of TIC and microbial biomass.

|  | **Groups** | **t** | **P(MC)** | **perms** |
| --- | --- | --- | --- | --- |
| **TIC** | Summer, Autumn | 0.291 | 0.778 | 10 |
|  | Summer, Winter | 3.700 | **0.027** | 10 |
|  | Summer, Spring | 0.645 | 0.561 | 10 |
|  | Autumn, Winter | 0.791 | 0.472 | 10 |
|  | Autumn, Spring | 0.148 | 0.890 | 10 |
|  | Winter, Spring | 0.812 | 0.478 | 10 |
| **Biomass** | Summer, Autumn | 1.266 | 0.276 | 10 |
|  | Summer, Winter | 1.434 | 0.216 | 10 |
|  | Summer, Spring | 0.204 | 0.854 | 10 |
|  | Autumn, Winter | 0.793 | 0.475 | 10 |
|  | Autumn, Spring | 1.076 | 0.363 | 10 |
|  | Winter, Spring | 0.656 | 0.540 | 10 |

Supplemental table S5. Statistical test results from pairwise PERMANOVA conducted for mineralization, assimilation, and decomposition rates of PE, PS, and plant litter to detect differences between seasons. Groups: Sum=summer, Aut=autumn, Win=winter, Spr=spring.

|  |  | **PE** |  |  | **PS** |  |  | **Plant litter** | |  |
| --- | --- | --- | --- | --- | --- | --- | --- | --- | --- | --- |
|  | **Groups** | **t** | **P(MC)** | **perms** | **t** | **P(MC)** | **perms** | **t** | **P(MC)** | **perms** |
| **Mineralization** | Sum, Aut | 0.060 | 0.953 | 10 | 5.551 | 0.006** | 10 | 25.376 | 0.001*** | 10 |
|  | Sum, Win | 0.539 | 0.596 | 10 | 6.902 | 0.002** | 10 | 30.102 | 0.001*** | 10 |
|  | Sum, Spr | 2.394 | 0.064 | 10 | 5.248 | 0.007** | 10 | 7.808 | 0.003** | 10 |
|  | Aut, Win | 0.449 | 0.671 | 10 | 0.631 | 0.588 | 10 | 4.324 | 0.014* | 10 |
|  | Aut, Spr | 1.292 | 0.273 | 10 | 0.496 | 0.629 | 10 | 2.679 | 0.057 | 10 |
|  | Win, Spr | 0.904 | 0.450 | 10 | 1.263 | 0.277 | 10 | 4.428 | 0.011* | 10 |
| **Assimilation** | Sum, Aut | 0.165 | 0.893 | 2 | 0.315 | 0.775 | 10 | 0.284 | 0.807 | 10 |
|  | Sum, Win | 0.801 | 0.443 | 2 | 0.565 | 0.592 | 10 | 2.039 | 0.109 | 10 |
|  | Sum, Spr | 0.935 | 0.417 | 2 | 0.692 | 0.540 | 10 | 0.191 | 0.845 | 10 |
|  | Aut, Win | 0.745 | 0.474 | 2 | 0.067 | 0.947 | 10 | 1.194 | 0.272 | 10 |
|  | Aut, Spr | 0.917 | 0.412 | 2 | 0.308 | 0.753 | 10 | 0.342 | 0.759 | 10 |
|  | Win, Spr | 0.622 | 0.559 | 2 | 0.339 | 0.762 | 10 | 1.911 | 0.127 | 10 |
| **Decomposition** | Sum, Aut | 0.06328 | 0.954 | 10 | 5.5609 | 0.011* | 10 | 30.822 | 0.001*** | 10 |
|  | Sum, Win | 0.4507 | 0.683 | 10 | 6.1869 | 0.005** | 10 | 29.786 | 0.001*** | 10 |
|  | Sum, Spr | 2.0326 | 0.112 | 10 | 5.3704 | 0.005** | 10 | 8.2494 | 0.002** | 10 |
|  | Aut, Win | 0.396 | 0.721 | 10 | 0.72044 | 0.511 | 10 | 4.5641 | 0.014* | 10 |
|  | Aut, Spr | 1.122 | 0.354 | 10 | 0.53296 | 0.613 | 10 | 2.8513 | 0.043* | 10 |
|  | Win, Spr | 0.69004 | 0.515 | 10 | 1.3409 | 0.251 | 10 | 4.4132 | 0.009** | 10 |
| **BGE** | Sum, Aut | 0.016 | 0.983 | 2 | 0.925 | 0.432 | 10 | 1.656 | 0.159 | 10 |
|  | Sum, Win | 0.831 | 0.442 | 4 | 3.243 | 0.035* | 10 | 4.282 | 0.017* | 10 |
|  | Sum, Spr | 0.938 | 0.388 | 2 | 0.390 | 0.743 | 10 | 1.421 | 0.218 | 10 |
|  | Aut, Win | 0.825 | 0.474 | 4 | 0.083 | 0.933 | 10 | 1.924 | 0.111 | 10 |
|  | Aut, Spr | 0.937 | 0.392 | 2 | 0.713 | 0.542 | 10 | 0.840 | 0.444 | 10 |
|  | Win, Spr | 0.712 | 0.509 | 4 | 1.442 | 0.222 | 10 | 3.227 | 0.033* | 10 |

Supplemental table S6. Statistical test results from pairwise PERMANOVA conducted for ^13^C-values of specific PLFAs in PE, PS, and plant litter treatment in comparison to control. *p<0.05,**p≤0.01, ***p≤0.001.

|  |  |  |  |  |  |  |  |  |  |  |  |  |  |
| --- | --- | --- | --- | --- | --- | --- | --- | --- | --- | --- | --- | --- | --- |
|  |  | **Summer** | |  | **Autumn** | |  | **Winter** | |  | **Spring** | |  |
|  |  | **t** | **P(MC)** | **perms** | **t** | **P(MC)** | **perms** | **t** | **P(MC)** | **perms** | **t** | **P(MC)** | **perms** |
| **PE** | **i15** | 2.54 | 0.013* | 997 | 0.01 | 0.985 | 990 | 1.82 | 0.081 | 32 | 5.67 | 0.001*** | 998 |
|  | **a15** | 0.85 | 0.417 | 126 | 0.01 | 0.994 | 985 | 2.41 | 0.02* | 960 | 4.99 | 0.001*** | 997 |
|  | **15:0** | 1.02 | 0.338 | 422 | 1.13 | 0.26 | 981 | 1.41 | 0.176 | 127 | 3.97 | 0.001*** | 994 |
|  | **16:1ꞷ7** | 3.28 | 0.005** | 997 | 0.76 | 0.473 | 994 | 3.60 | 0.003** | 998 | 2.33 | 0.029* | 999 |
|  | **17:0** | nd | 0.335 | nd | 1.19 | 0.251 | 992 | 0.37 | 0.7 | 64 | 2.59 | 0.016* | 989 |
|  | **18:1ꞷ9** | 3.83 | 0.002** | 996 | 0.75 | 0.466 | 996 | 5.70 | 0.001*** | 978 | 1.68 | 0.118 | 997 |
|  | **18:1ꞷ7** | 3.54 | 0.004** | 996 | 0.36 | 0.713 | 995 | 0.13 | 0.899 | 997 | 1.82 | 0.079 | 995 |
| **PS** | **i15** | 19.48 | 0.001*** | 995 | 0.25 | 0.803 | 987 | 2.77 | 0.012* | 873 | 3.08 | 0.009** | 999 |
|  | **a15** | 0.70 | 0.502 | 426 | 0.69 | 0.5 | 950 | 2.36 | 0.017* | 999 | 1.26 | 0.202 | 996 |
|  | **15:0** | 0.66 | 0.521 | 864 | 0.15 | 0.871 | 995 | 2.19 | 0.047* | 975 | 1.90 | 0.069 | 990 |
|  | **16:1ꞷ7** | 11.87 | 0.001*** | 996 | 2.60 | 0.023* | 995 | 0.23 | 0.838 | 998 | 4.65 | 0.001*** | 997 |
|  | **17:0** | 0.61 | 0.568 | 64 | 1.66 | 0.115 | 959 | 1.12 | 0.286 | 606 | 1.14 | 0.274 | 990 |
|  | **18:1ꞷ9** | 4.58 | 0.001*** | 999 | 1.03 | 0.316 | 990 | 1.74 | 0.087 | 996 | 1.70 | 0.11 | 989 |
|  | **18:1ꞷ7** | 6.55 | 0.001*** | 997 | 2.09 | 0.056 | 997 | 2.39 | 0.026* | 998 | 4.95 | 0.001*** | 986 |
| **Plant** | **i15** | 10.93 | 0.001*** | 997 | 3.35 | 0.003** | 968 | 10.70 | 0.001*** | 988 | 11.43 | 0.001*** | 995 |
| **litter** | **a15** | 10.72 | 0.001*** | 991 | 3.82 | 0.001*** | 967 | 9.74 | 0.001*** | 994 | 8.83 | 0.001*** | 996 |
|  | **15:0** | 4.57 | 0.001*** | 946 | 1.77 | 0.099 | 976 | 7.12 | 0.001*** | 989 | 3.67 | 0.004** | 998 |
|  | **16:1ꞷ7** | 11.30 | 0.001*** | 998 | 5.36 | 0.001*** | 992 | 7.26 | 0.001*** | 993 | 8.37 | 0.001*** | 996 |
|  | **17:0** | 1.14 | 0.263 | 64 | 2.31 | 0.033* | 972 | 0.87 | 0.406 | 127 | 1.32 | 0.212 | 983 |
|  | **18:1ꞷ9** | 2.13 | 0.047 | 997 | 2.10 | 0.047* | 994 | 3.73 | 0.002** | 995 | 2.88 | 0.004** | 998 |
|  | **18:1ꞷ7** | 8.37 | 0.001*** | 996 | 3.15 | 0.008** | 994 | 6.01 | 0.001*** | 998 | 10.50 | 0.001*** | 994 |

Supplemental table S7. Microbial communities (>0.5 % of all OTUs) differed significantly between each four seasons at the genus and class levels (pair-wise PERMANOVA).

|  | **Genus** |  |  | **Class** |  |  |
| --- | --- | --- | --- | --- | --- | --- |
| **Groups** | **t** | **P(MC)** | **Unique perms** | **t** | **P(MC)** | **Unique perms** |
| Summer, Autumn | 7.95 | 0.001 | 999 | 10.5 | 0.001 | 999 |
| Summer, Winter | 10.32 | 0.001 | 998 | 14 | 0.001 | 998 |
| Summer, Spring | 7.74 | 0.001 | 999 | 8.3 | 0.001 | 998 |
| Autumn, Winter | 6.08 | 0.001 | 998 | 6.5 | 0.001 | 999 |
| Autumn, Spring | 5.28 | 0.001 | 998 | 4.8 | 0.001 | 999 |
| Winter, Spring | 6.32 | 0.001 | 999 | 7.6 | 0.001 | 999 |

Supplemental table S8. Microbial communities (>0.5 % of all OTUs) differed significantly between three lake water at the genus and class levels (pair-wise PERMANOVA).

|  |  |  |  |  |  |  |
| --- | --- | --- | --- | --- | --- | --- |
|  | **Genus** |  |  | **Class** |  |  |
| **Groups** | **t** | **P(MC)** | **Unique perms** | **t** | **P(MC)** | **Unique perms** |
| Haukijärvi, Majajärvi | 3.59 | 0.001 | 999 | 2.97 | 0.001 | 998 |
| Haukijärvi, Nimetön | 3.02 | 0.001 | 999 | 3.62 | 0.001 | 998 |
| Majajärvi, Nimetön | 3.89 | 0.001 | 998 | 3.74 | 0.001 | 999 |

Supplemental table S9. SIMPER analysis (cumulative contribution % > 70 %) showing the effects of plant litter addition on microbial community structures at the class level. Suggested plant litter decomposer classes (increased abundance in plant litter treatment) have been bolded.

|  |  | **Control** | **Plant litter** |  |  | |  | | |  |
| --- | --- | --- | --- | --- | --- | --- | --- | --- | --- | --- |
|  | Class | Av.Abund | Av.Abund | Av.Diss | Contrib% | Cum.% | |  |  |  |
| **Summer** | **Deltaproteobacteria** | 3.06 | 5.74 | 4.08 | 13.33 | 13.33 | |  |  |  |
|  | Acidobacteriia | 4.24 | 2.55 | 2.54 | 8.3 | 21.63 | |  |  |  |
|  | Alphaproteobacteria | 3.39 | 1.94 | 2.19 | 7.15 | 28.79 | |  |  |  |
|  | **Verrucomicrobiae** | 3.55 | 4.49 | 2.11 | 6.89 | 35.68 | |  |  |  |
|  | Planctomycetacia | 3.02 | 1.91 | 1.91 | 6.24 | 41.92 | |  |  |  |
|  | Elusimicrobia | 1.38 | 0.56 | 1.72 | 5.63 | 47.54 | |  |  |  |
|  | Phycisphaerae | 1.86 | 1.14 | 1.68 | 5.5 | 53.04 | |  |  |  |
|  | Hydrogenedentia | 1.19 | 0.88 | 1.17 | 3.81 | 56.85 | |  |  |  |
|  | **Gammaproteobacteria** | 3.53 | 4.24 | 1.16 | 3.77 | 60.62 | |  |  |  |
|  | Babeliae | 0.46 | 0.51 | 1.09 | 3.54 | 64.17 | |  |  |  |
|  | TK10 | 0.72 | 0.16 | 1.06 | 3.46 | 67.62 | |  |  |  |
|  | Fimbriimonadia | 1.11 | 0.88 | 1.04 | 3.39 | 71.01 | |  |  |  |
| **Autumn** | **Verrucomicrobiae** | 2.03 | 4 | 6.16 | 21.14 | 21.14 | |  |  |  |
|  | Planctomycetacia | 1.35 | 0.33 | 2.55 | 8.77 | 29.91 | |  |  |  |
|  | Alphaproteobacteria | 4.62 | 3.59 | 2.45 | 8.41 | 38.32 | |  |  |  |
|  | Fimbriimonadia | 1.08 | 1.04 | 2.28 | 7.84 | 46.16 | |  |  |  |
|  | Babeliae | 1.18 | 0.8 | 2.26 | 7.76 | 53.92 | |  |  |  |
|  | Gammaproteobacteria | 7.46 | 7.29 | 2.07 | 7.11 | 61.03 | |  |  |  |
|  | Chloroflexia | 1.2 | 0.96 | 1.73 | 5.92 | 66.95 | |  |  |  |
|  | Bacteroidia | 1.56 | 0.91 | 1.71 | 5.88 | 72.84 | |  |  |  |
| **Winter** | **Verrucomicrobiae** | 3.16 | 3.33 | 5.31 | 21.77 | 21.77 | | |  |  |
|  | **Bacteroidia** | 0.98 | 2.21 | 3.5 | 14.32 | 36.09 | | |  |  |
|  | **Deltaproteobacteria** | 0.66 | 1.05 | 3.17 | 12.99 | 49.08 | | |  |  |
|  | Gammaproteobacteria | 8.28 | 7.95 | 2.39 | 9.8 | 58.88 | | |  |  |
|  | Lentisphaeria | 0.74 | 0.49 | 2.07 | 8.47 | 67.35 | | |  |  |
|  | Actinobacteria | 1.77 | 1.67 | 1.67 | 6.84 | 74.19 | | |  |  |
| **Spring** | **vadinHA49** | 0 | 2.21 | 4.23 | 13.33 | 13.33 | | |  |  |
|  | **Verrucomicrobiae** | 3.8 | 5.86 | 4.08 | 12.86 | 26.19 | | |  |  |
|  | **Fibrobacteria** | 0 | 1.7 | 3.12 | 9.85 | 36.04 | | |  |  |
|  | Gammaproteobacteria | 6.18 | 4.77 | 2.86 | 9.03 | 45.07 | | |  |  |
|  | Alphaproteobacteria | 4.69 | 3.4 | 2.74 | 8.63 | 53.7 | | |  |  |
|  | **Deltaproteobacteria** | 1.74 | 2.6 | 1.9 | 5.99 | 59.7 | | |  |  |
|  | Fimbriimonadia | 1.78 | 0.88 | 1.82 | 5.74 | 65.43 | | |  |  |
|  | Acidobacteriia | 1.72 | 1.29 | 1.77 | 5.59 | 71.02 | | |  |  |

Supplemental table S10. Pearson correlations (NMDS1>0.6) of bacterial genera correlating towards lake water with the fastest decomposition rate in summer. Correl indicates the Pearson correlation of each genus separately with the decomposition rate of PS, and R^2^ is the coefficient of determination. In summer, 13 genera correlated towards lake waters with higher decomposition rates. Among these, 6 genera correlated with a decomposition rate (Pearson correlation and R^2^ > 0.6).

| **Genus** | **NMDS1** | **NMDS2** | **Correl** | **R^2^** |
| --- | --- | --- | --- | --- |
| uncultured_Micropepsaceae | 0.92 | 0.32 | 0.95 | 0.91 |
| Rhodovastum | 0.88 | 0.32 | 0.82 | 0.68 |
| Porticoccus | 0.87 | 0.18 | 0.89 | 0.79 |
| Candidatus Solibacter | 0.86 | 0.44 | 0.86 | 0.75 |
| Iamia | 0.82 | 0.07 | 0.73 | 0.53 |
| uncultured_Rhodobacteraceae | 0.79 | 0.34 | 0.87 | 0.75 |
| uncultured_Steroidobacteraceae | 0.77 | 0.09 | 0.68 | 0.46 |
| uncultured_Roseiflexaceae | 0.76 | -0.05 | 0.63 | 0.40 |
| uncultured_IMCC26257 | 0.75 | -0.05 | 0.65 | 0.42 |
| uncultured_KF-JG30-B3 | 0.74 | 0.45 | 0.75 | 0.56 |
| uncultured_Lineage IV | 0.73 | 0.08 | 0.74 | 0.55 |
| Zoogloea | 0.71 | 0.63 | 0.80 | 0.64 |
| uncultured_Isosphaeraceae | 0.61 | -0.58 | 0.46 | 0.21 |

Supplemental table S11. Pearson correlations (NMDS1<0.6) of bacterial genera correlating towards lake water with the fastest decomposition rate in autumn. Correl indicates the Pearson correlation of each genus separately with the decomposition rate of PS, and R^2^ is the coefficient of determination. In autumn, 11 genera correlated towards lake water microbiomes where PS was decomposed at the fastest rate. Among these taxa, 6 genera correlated straightly with the decomposition rate.

| **Genus** | **NMDS1** | **NMDS2** | **CORREL** | **R^2^** |
| --- | --- | --- | --- | --- |
| uncultured_Beijerinckaceae | -0.99 | -0.06 | 0.68 | 0.46 |
| uncultured_Burkholderiaceae | -0.97 | -0.07 | 0.70 | 0.49 |
| Aquabacterium | -0.96 | 0.12 | 0.89 | 0.79 |
| Candidatus Methylopumilus | -0.95 | 0.21 | 0.81 | 0.65 |
| Caulobacter | -0.91 | 0.28 | 0.80 | 0.64 |
| uncultured_Beijerinckiaceae | -0.88 | 0.42 | 0.95 | 0.91 |
| Beijerinckia | -0.88 | 0.37 | 0.92 | 0.85 |
| uncultured_TRA3-20 | -0.83 | 0.52 | 0.95 | 0.90 |
| Opitutus | -0.76 | 0.58 | 0.91 | 0.83 |
| uncultured_Micropepsaceae | -0.65 | 0.70 | 0.90 | 0.81 |
| Candidatus Solibacter | -0.61 | 0.65 | 0.75 | 0.56 |

Supplemental table S12. Pearson correlations (NMDS1<0.6) of bacterial genera correlating towards lake water with the fastest decomposition rate in spring. Correl indicates the Pearson correlation of each genus separately with the decomposition rate of PS, and R^2^ is the coefficient of determination. In spring, 23 genera correlated towards a fast decomposition rate. Among these, 6 genera correlated with a decomposition rate Pearson correlation > 0.6, R^2^ > 0.5).

| **Genus** | **NMDS1** | **NMDS2** | **Correl** | **R^2^** |
| --- | --- | --- | --- | --- |
| Candidatus Solibacter | -0.98 | -0.05 | 0.74 | 0.55 |
| uncultured_Beijerinckaceae | -0.97 | 0.15 | 0.76 | 0.57 |
| Methylotenera | -0.95 | -0.03 | 0.71 | 0.51 |
| uncultured_Sneathiellaceae | -0.91 | 0.22 | 0.76 | 0.57 |
| Methylorosula | -0.90 | -0.01 | 0.65 | 0.43 |
| uncultured_Pedosphaeraceae | -0.90 | 0.24 | 0.77 | 0.59 |
| uncultured_TRA3-20 | -0.89 | 0.05 | 0.59 | 0.35 |
| uncultured_Micropepsaceae | -0.86 | -0.03 | 0.52 | 0.27 |
| GKS98 freshwater group | -0.80 | -0.18 | 0.60 | 0.35 |
| Polynucleobacter | -0.80 | 0.06 | 0.63 | 0.40 |
| GOUTA6 | -0.78 | 0.32 | 0.72 | 0.52 |
| FukuN57 | -0.77 | 0.16 | 0.55 | 0.30 |
| Aquabacterium | -0.76 | -0.04 | 0.44 | 0.19 |
| uncultured_Beijerinckiaceae | -0.76 | -0.09 | 0.41 | 0.16 |
| Legionella | -0.70 | 0.02 | 0.46 | 0.21 |
| Rhodovastum | -0.70 | -0.17 | 0.36 | 0.13 |
| Limnohabitans | -0.69 | 0.24 | 0.62 | 0.38 |
| Polaromonas | -0.68 | 0.02 | 0.55 | 0.30 |
| Beijerinckia | -0.67 | -0.09 | 0.36 | 0.13 |
| uncultured_Nitrosomonadaceae | -0.65 | 0.38 | 0.66 | 0.44 |
| Opitutus | -0.65 | -0.08 | 0.30 | 0.09 |
| uncultured_Acidimicrobiia | -0.61 | -0.04 | 0.28 | 0.08 |
| Caulobacter | -0.61 | -0.40 | 0.29 | 0.08 |

Supplemental table S13. Pearson correlations (NMDS1<0.6) of bacterial genera correlating towards lake water with the fastest decomposition rate in winter. Comparison was made between lake Haukijärvi (the lowest rate) and the lake Majajärvi lake (higher rate) microbiome. Correl indicates the Pearson correlation of each genus separately with the decomposition rate of PS, and R^2^ is the coefficient of determination. 15 genera correlated towards faster PS decomposition. Among these, relative abundance of 13 genera correlated straightly with decomposition rate of PS (Pearson correlation >0.8, R^2^>0.6).

| **Genus** | **NMDS1** | **NMDS2** | **Correl** | **R^2^** |
| --- | --- | --- | --- | --- |
| Pseudomonas | -1.00 | -0.03 | 0.95 | 0.89 |
| Nitrosospira | -0.97 | -0.07 | 0.93 | 0.87 |
| Iodobacter | -0.97 | 0.22 | 0.87 | 0.75 |
| Bryobacter | -0.96 | -0.12 | 1.00 | 0.99 |
| uncultured_Rhodospirillales | -0.96 | -0.14 | 0.93 | 0.87 |
| uncultured_Gaiellales | -0.96 | -0.12 | 0.96 | 0.92 |
| uncultured_Gemmatimonadaceae | -0.95 | -0.08 | 0.88 | 0.78 |
| uncultured_Pedosphaeraceae | -0.95 | -0.23 | 0.98 | 0.95 |
| Herminiimonas | -0.93 | 0.00 | 0.85 | 0.72 |
| GOUTA6 | -0.92 | 0.15 | 0.84 | 0.71 |
| Opitutus | -0.90 | -0.39 | 0.96 | 0.91 |
| Caulobacter | -0.86 | 0.24 | 0.74 | 0.55 |
| uncultured_Micropepsaceae | -0.85 | -0.40 | 0.97 | 0.93 |
| Phenylobacterium | -0.84 | -0.20 | 0.91 | 0.83 |
| Undibacterium | -0.68 | 0.15 | 0.48 | 0.23 |
|  |  |  |  |  |

Supplemental table S14. Pearson correlations (NMDS1<0.6) of bacterial genera correlating towards lake water with the fastest decomposition rate in winter. Comparison was made between lake Haukijärvi (the lowest rate) and the lake Nimetön (the highest rate) microbiome. Correl indicates the Pearson correlation of each genus separately with the decomposition rate of PS, and R^2^ is the coefficient of determination. 12 genera correlated towards lake water with faster PS decomposition. Among these, relative abundance of 9 genera correlated straightly with decomposition rate of PS (Pearson correlation >0.8, R^2^>0.6).

| **Genus** | **NMDS1** | **NMDS2** | **Correl** | **R^2^** |
| --- | --- | --- | --- | --- |
| Candidatus Methylopumilus | -0.99 | -0.06 | 0.97 | 0.94 |
| Methylotenera | -0.97 | 0.01 | 0.97 | 0.94 |
| uncultured_Victivallales | -0.97 | 0.06 | 0.97 | 0.94 |
| RS62 marine group | -0.96 | 0.03 | 0.98 | 0.96 |
| Opitutus | -0.94 | 0.05 | 0.94 | 0.88 |
| uncultured_Beijerinckiaceae | -0.93 | 0.12 | 0.92 | 0.85 |
| Luteolibacter | -0.89 | -0.04 | 0.92 | 0.85 |
| uncultured_Acidimicrobiia | -0.85 | 0.01 | 0.86 | 0.74 |
| FukuN57 | -0.84 | -0.27 | 0.81 | 0.66 |
| GKS98 freshwater group | -0.80 | -0.50 | 0.75 | 0.56 |
| Limnohabitans | -0.75 | 0.22 | 0.76 | 0.58 |
| Rhodobacter | -0.69 | -0.49 | 0.62 | 0.38 |
